# Effects of Sugarcane Leaf Return and Fertilizer Reduction on Maize Growth, Yield and Soil Properties in Red Soil

**DOI:** 10.3390/plants12051029

**Published:** 2023-02-24

**Authors:** Yufeng Liu, Yumo Tan, Dan Liang, Chengruo Pei, Zhenhua Zhang

**Affiliations:** 1 Agricultural Resources and Environmental Research Institute, Guangxi Academy of Agricultural Sciences/Guangxi Key Laboratory of Arable Land Conservation, Nanning 530007, China; 2 Guangxi Vocational College of Water Resources and Electric Power, Nanning 530023, China; 3 Institute of Jiangsu Coastal Agricultural Sciences, Yancheng 224002, China; 4 School of Agriculture and Environment, The University of Western Australia, Crawley, WA 6009, Australia

**Keywords:** fertilizer reduction, maize growth and yield, red soil, soil properties, sugarcane leaf return

## Abstract

In order to make better use of the vast sugarcane leaf straw resources and reduce the overuse of chemical fertilizers in the subtropical red soil region of Guangxi, this study aimed to determine the effects of sugarcane leaf return (SLR) and fertilizer reduction (FR) on maize growth, yield component and yield, and soil properties. A pot experiment with three SLR amounts (full SLR (FS), 120 g/pot; half SLR (HS), 60 g/pot; and no SLR (NS) with three FR levels including full fertilizer (FF), 4.50 g N/pot, 3.00 g P_2_O_5_/pot, and 4.50 g K_2_O/pot; half fertilizer (HF), 2.25 g N/pot, 1.50 g P_2_O_5_/pot, and 2.25 g K_2_O/pot; and no fertilizer (NF)), without nitrogen, phosphorous, and potassium added, was conducted to assess the effects of different SLR amounts and chemical FR levels on maize growth, yield, and soil properties. Compared with no sugarcane leaf return and the no-fertilizer treatment (CK), SLR and FR could increase maize plant height, stalk diameter, number of fully developed maize plant leaves, total leaf area and chlorophyll content, soil alkali–hydrolyzable nitrogen (AN), available phosphorus (AP), available potassium (AK), soil organic matter (SOM), and electrical conductivity (EC). The maize yield component factors of FS and HS were higher in NF treatment than those in NS treatment. The relative increase rate of treatments retained FF/NF and HF/NF under FS or HS condition on 1000 kernel weight, ear diameter, plant air-dried weight, ear height, and yield than that under NS condition. FSHF had not only the largest plant air-dried weight but also the highest maize yield (3225.08 kg/hm^2^) among nine treatment combinations. The effects of SLR on maize growth and yield and soil properties were lower than those of FR. SLR and FR combined treatment did not affect maize growth but affected maize yield significantly. Soil properties improved more with SLR + FR treatment than with SLR or FR application alone. The plant height, stalk diameter, number of fully developed maize plant leaves, and total leaf area, as well as AN, AP, AK, SOM, and EC levels in soil, were enhanced by SLR and FR incorporation. The experimental results indicated that applying reasonable FR combined with SLR increased AN, AP, AK, SOM, and EC, which improved maize growth and yield and enhanced soil properties in red soil. Hence, FSHF might be a suitable combination of SLR and FR.

## 1. Introduction

Sugarcane is cultivated widely in southern China, and China is the fourth-largest sugarcane producer in the world, following Brazil, India, and Thailand [1]. Guangxi Zhuang Autonomous Region (Guangxi) is the biggest sugarcane-producing province in China, contributing 60–70% to the nationwide sugarcane production [2,3]. About 8.9–14.8 million tons (Mt) of sugarcane leaf residues are produced in Guangxi annually. The N, P_2_O_5_, K_2_O and organic C content of sugarcane leaf residues were recorded as 15 g/kg, 2.3 g/kg, 5.5 g/kg and 500 g/kg [4,5,6], respectively. It follows that it should contain 180,000 t, 27,600 t, 66,000 t and 6.0 million t of N, P_2_O_5_, K_2_O and organic C per year respectively, which is equivalent to 391,000 t, 230,000 t and 110,000 t of urea (N46%), calcium superphosphate (P_2_O_5_ 12%), potash chloride (K_2_O 60%) fertilizers and 15.0 million t organic fertilizer (organic content 40%), respectively. Most sugarcane leaf residues are discarded or burned on the spot in the harvest season, which is the main way to get rid of leaf residues in Guangxi, because of the lack of effective resources and industrial utilization. However, discard or burning of sugarcane leaves could cause serious problems such as air pollution, greenhouse gas emission [7], nutrient loss [8], fire and traffic accidents, and so on. If sugarcane leaves residues was burned in Guangxi, approximately 24 million t CO_2_ and noxious gas would be released into atmosphere per year. The rational and effective use of sugarcane leaves resources is becoming an inevitable requirement for the sustainable development of the Guangxi sugarcane industry.

Sugarcane leaf return (SLR) to the field has been recognized and implemented not only as a simple, effective, and environmentally friendly practice, but also as a technique that can enhance soil organic carbon levels and improve crop production [9,10]. Since the 1980s, SLR has been researched in sugarcane production areas of southern China, where many benefits of SLR have been reported [11], such as increasing sugarcane yield [9], reducing air pollution and greenhouse gas emission [12], increasing soil carbon stocks [13] and soil microbial activity [14], regulating soil temperature and water losses [15], enhancing nutrient cycling [16], and preserving soil structure [17]. Therefore, the transition from burning and discarding sugarcane residues to SLR to field cultivation system has significantly preserved the ecological environment and improved the sustainability of the sugarcane production chain and soil health. On the contrary, some adverse impacts of SLR have been reported, such as a reduction in the numbers of perennial sugarcane root sprouting and yield [18,19], increasing incidence of pests and diseases [20], difficulties in mechanized cultivation [21], and so on. Other studies reported that the long-term straw return alone on crop yield was similar to no fertilizer application [22,23]. Straw contains a large amount of cellulose, hemicellulose, lignin, and other carbonaceous substances, with a high C/N ratio [24,25,26] and a relatively low nutrient content. This leads to the competition of nutrient sharing between soil microbes and crops during the straw decomposition process, affecting the growth and yield of crops. A certain amount of chemical fertilizers should be added to sugarcane leaf residues to promote the decomposition of the sugarcane straw and increase the content of inorganic nutrients in the soil so as to control the C/N ratio within a reasonable range [27]. Long-term field experiments have proved that a combination of straw return and fertilizer can help achieve crop yield in a similar way to chemical fertilizer alone. However, the benefits of SLR and FR combined treatment are still unclear.

Fertilization, as a common and global agricultural management strategy, is not only a tool for enhancing crop productivity but also for improving the health of the soil ecological environment [28,29]. China has been the world’s leading consumer of chemical fertilizers since 1985 [30]. From 1980 (9.3 Mt) to 2012 (24 Mt), a 158% increase in fertilizer use was associated with a 70% increase in China’s crop yield (321–547 Mt) [31], clearly indicating a disproportionately low increase in crop yield compared with the increase in the use of chemical fertilizers. In 2015, the average chemical fertilizer consumption was 446 kg/hm^2^ in China, and the average chemical fertilizer consumption per square hectometer arable land in China was about 3.6 times higher than the world average level during the corresponding period [32]. The long-term overuse of chemical fertilizers in the croplands had numerous negative environmental impacts, such as greenhouse gas excess emission [33], soil degradation [34], soil acidification [35], agricultural non-point source pollution [10], decrease in soil biodiversity [36], and so on. Optimizing fertilization management practices to minimize adverse environmental impacts while increasing crop yield is vital. Recent studies have reported the aforementioned adverse impacts of chemical fertilizer overuse in the absence of organic material application in farming. These adverse impacts on soil properties jeopardized the output and sustainability of the sugarcane industry system in Guangxi. It is necessary to reduce chemical fertilizer consumption to better achieve soil health and a sustainable agroecosystem [37]. Therefore, exploring the impact of fertilizer reduction (FR) and SLR on crop growth and yield and soil properties is crucial.

Red soil is the main and typical soil type in southern China [38,39], and in Guangxi, it covers about 107.44 × 10^3^ km^2^, which accounts for 45.2% of the total land area [40]. In Guangxi, 80% of sugarcane and other staple crops are cultivated in the subtropical red soil region [41]. Studies on the impacts of SLR and FR combined treatment on crop growth, yield, and soil properties are limited. The red soil in subtropical region was eroded easily by rainfall and high temperature. The mineralization and decomposition of organic matter in red soil was accelerated. On the one hand, lots of organic fertilizer had been used to maintain soil fertility and avoid soil acidification; on the other hand, sugarcane leaf residues, which mainly contain organic matter and mineral nutrition, were burned and discarded on the spot. Hence, studying crop growth, yield, and soil properties in red soil areas is of paramount importance; facilitating the research into and popularization of sugarcane leaf return and fertilizer reduction in Guangxi red soil would bring remarkable social, economy and ecological benefits. Further, studies on how to combine the use of fertilizers with SLR to enhance crop yields and promote soil fertility are lacking. Hence, this study selected Guangxi red soil to examine the influence of SLR and FR on maize growth, yield, and soil properties. This study aimed to (1) analyze and compare the effects of SLR and FR on maize growth and yield, (2) analyze and compare the effects of SLR and FR on Guangxi red soil properties, and (3) analyze the relationships between the soil properties, crop growth, and yield. This study also aimed to contribute to the comprehensive understanding of the proper use of sugarcane leaf resources in southern China.

## 2. Results

### 2.1. Maize Growth

**Figure 1:**
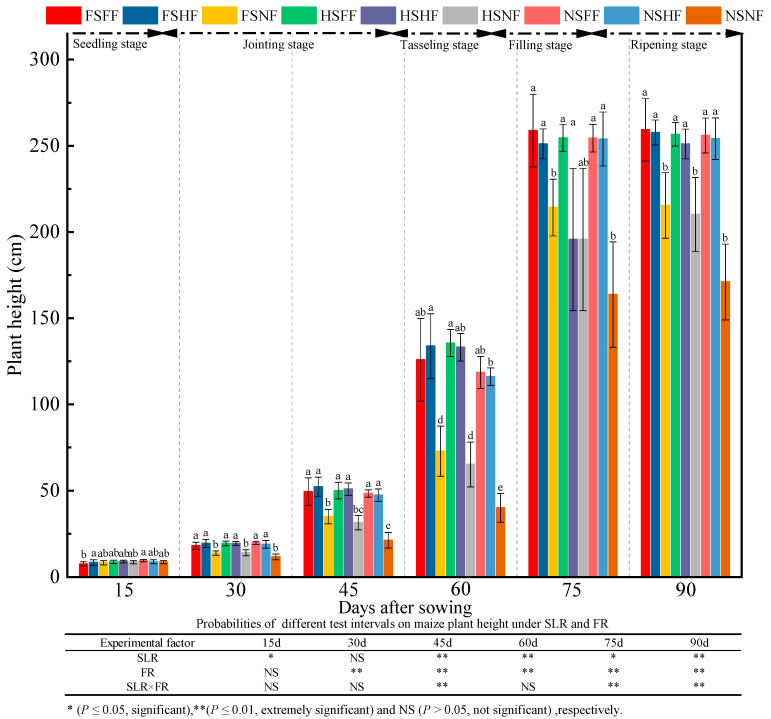
Effects of SLR and FR on plant height. Note: Bars represent means ± standard deviation (*n* = 3) with different letters indicating significant differences based on LSD (*p* ≤ 0.05). SLR, sugarcane leaf return; FR, fertilizer reduction. The symbols in the following tables and figures are the same as those appearing in this figure.

As shown in Figure 1, the average plant height after 15, 30, 45, 60, 75, and 90 days was 8.65, 17.25, 42.87, 104.57, 233.03, and 236.79 cm, with an average increase of 99.35%, 148.54%, 143.94%, 122.85% and 1.61%, respectively, in the plant height between two consecutive observation time points. The analysis results showed that the maize plant height increased slowly in the seedling stage (15 days), increased rapidly from 30 to 75 days, and peaked in the filling stage. It indicated a significant effect of SLR on plant height throughout the growth stage except seedling stage. The maize plant height was extremely significantly affected by FR at all observation time points except after 15 days (*p* < 0.01). The plant height significantly increased after 45, 75, and 90 days of combined SLR and FR treatment. In the seedling stage, a significant difference in height was not found between different treatments. After 30 days, the impacts of experimental treatments on plant height appeared gradually. Except in the seedling stage, the height of the maize under the FF and HF conditions was significantly higher than that under the NF condition (*p* < 0.05), especially under the NS condition. Except in the seedling stage, under the FS and HS condition, the average plant height increase rate under the FF and HF conditions was 37.02%, 52.68%, 92.14%, 24.13%, and 20.41% of that under the NF condition. However, the average increase rate of plant height under the FF and HF conditions compared with NF treatment was 65.37%, 124.80%, 192.74%, 55.23%, and 49.13%, respectively, under the NS condition. Therefore, the study indicated that the average increase in plant height without SLR was about two times higher than that with SLR. Under the FF and HF conditions, no statistically significant difference was found in plant height between FS and HS conditions. However, plant height under SLR condition was in the order FS > HS > NS.

**Figure 2:**
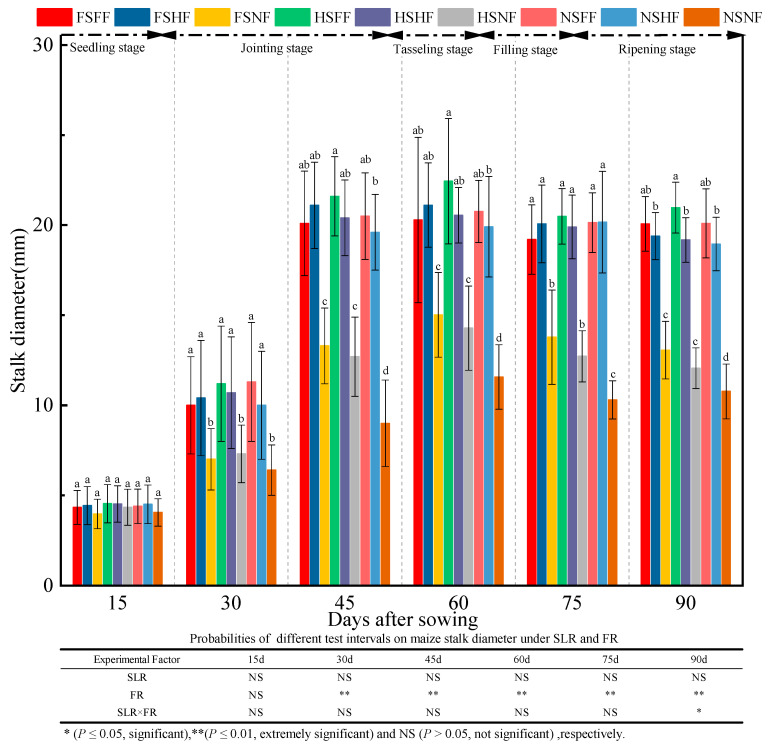
Effects of SLR and FR on stalk diameter. Note: Bars represent means ± standard deviation (*n* = 3) with different letters indicating significant differences based on LSD (*p* ≤ 0.05). SLR, sugarcane leaf return; FR, fertilizer reduction.

The changes in maize stalk diameter in the whole growth stage were as shown in Figure 2. Except after 15 days, the effect of FR on maize stem diameter was significant (*p* < 0.01). However, the interaction effect of SLR and FR combined treatment was just opposite. In the seedling stage, the variation in stalk diameter was insignificant in all nine treatment combinations (*p* > 0.05). After 15 days, the effect of experimental treatments on stalk diameter began to appear gradually. The stalk diameter of maize plants under FF and HF condition was highly significant than that under the NF condition (*p* < 0.05) after the seedling stage except after 15 days. Under the FF and HF conditions, a significant difference in the stalk diameter was not found between FS and HS treatments. Further, the stalk diameter after HSFF treatment was always more significant than that after HSHF treatment; however, the stalk diameter was more significant under FSFF treatment than under FSHF treatment after 90 days. Under the NF condition, the maize stalk diameter under FS and HS conditions was highly significant (*p* < 0.05) than that under the NS condition after the jointing stage. Meanwhile, the maize stalk diameter was lower throughout under the HS condition than under the FS condition. Under the FS and HS conditions, the average increase in stalk diameter in FF and HF vs. NF was 7.35%, 47.86%, 60.12%, 44.17%, 50.63%, and 58.77%, respectively. However, under the NS condition, the average increase rate in FF and HF vs. NF was 9.75%, 66.41%, 122.78%, 75.76%, 95.68%, and 81.29%, respectively.

**Figure 3:**
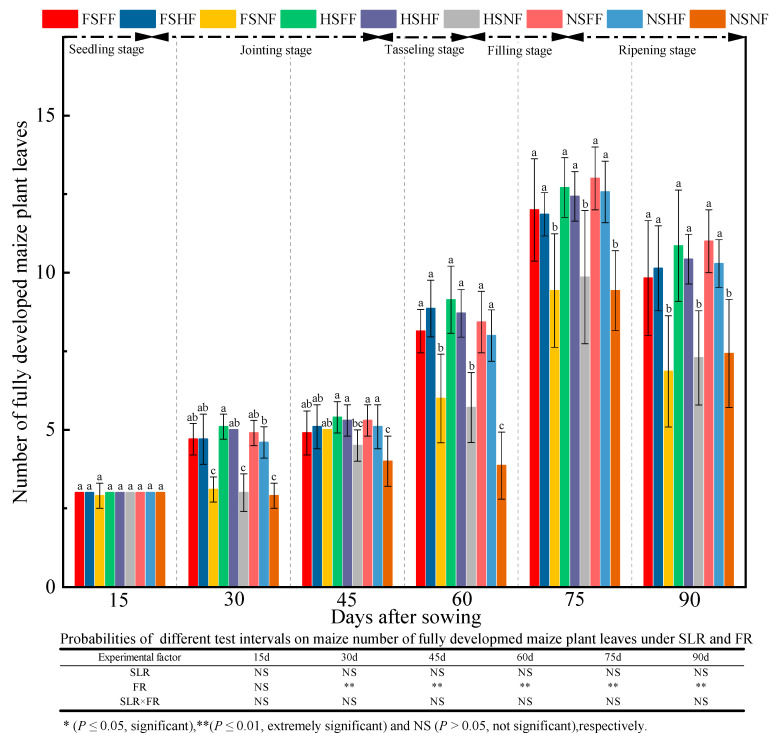
Effects of SLR and FR on the number of fully developed maize plant leaves. Note: Bars represent means ± standard deviation (*n* = 3) with different letters indicating significant differences based on LSD (*p* ≤ 0.05). SLR, sugarcane leaf return; FR, fertilizer reduction.

As shown in Figure 3, no significant difference was observed in the number of fully developed maize plant leaves among nine treatment combinations in the seedling stage. After the seedling stage, the impact of experimental treatment on the number of fully developed maize plant leaves started to appear gradually. The average number of fully developed maize plant leaves was 2.99, 4.22, 4.96, 7.43, 11.48, and 9.35 after 15, 30, 45, 60, 75, and 90 days, respectively. The number of fully developed maize plant leaves was significantly higher (*p* < 0.05) under the FF and HF conditions than that under the NF condition except after 15 days. Under NF condition, the number of fully developed maize plant leaves had SLR levels in the order FS > HS > NS in the jointing and tasseling stages. Under the FS and HS conditions, the average increase rate in the number of fully developed maize plant leaves under FF and HF vs. NF was 57.26%, 3.50%, 45.21%, and 29.90% after 30, 45, 60, and 75 days, respectively. Under the NS condition, the average increase rate in the number of fully developed maize plant leaves under FF and HF vs. NF was 63.79%, 30.00%, 112.82%, and 35.58%, respectively. The analysis results of the number of fully developed maize plant leaves in all treatments showed that FR had an extremely significant effect on the number of fully developed maize plant leaves (*p* < 0.01) except seedling stage, but the effects of SLR and combined treatment with SLR and FR were the opposite.

**Figure 4:**
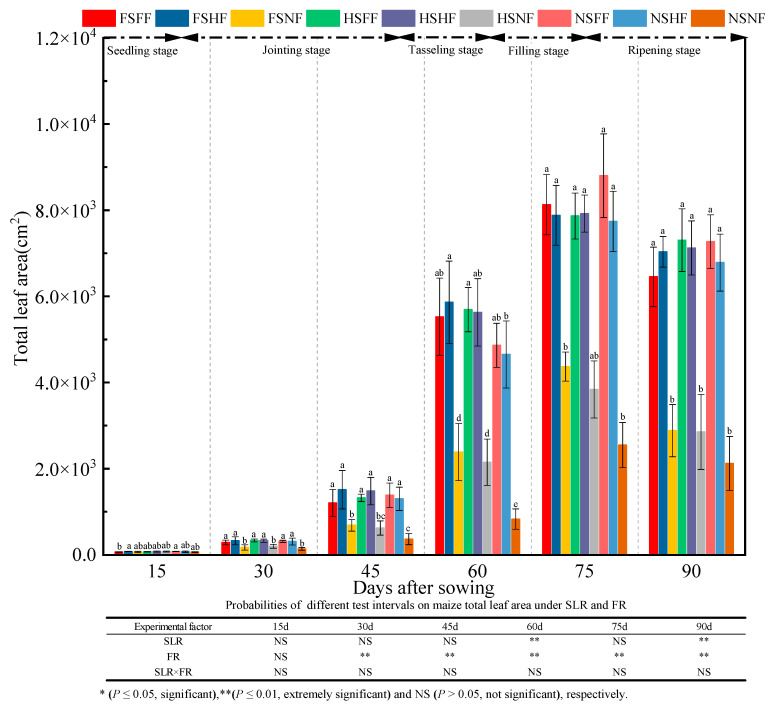
Effects of SLR and FR on total leaf area. Note: Bars represent means ± standard deviation (*n* = 3) with different letters indicating significant differences based on LSD (*p* ≤ 0.05). SLR, sugarcane leaf return; FR, fertilizer reduction.

The maize total leaf area in nine treatment combinations during the whole growth stage was as shown in Figure 4. The analysis results showed that treatments had extremely significant effects on the total leaf area, and the latter was affected by the SLR (except after 15 days) and FR (after 60 and 90 days) (*p* < 0.01). However, the interaction effect of SLR and FR combined treatment on the total leaf area was not significantly different. In the seedling stage, no significant difference was observed in the effect on the total leaf area in all nine treatment combinations. After the seedling stage, the total leaf area under the FF and HF conditions was more significantly affected (*p* < 0.05) than that under the NF (CK) condition. Under the NF condition, the total leaf area of the maize plants among the three SLR treatment levels was in the order FS > HS > NS. A significant difference (*p* < 0.05) was observed on total leaf area among the three SLR treatment levels after 40, 60, and 75 days of sowing. The average total leaf area was 75.69, 271.76, 1097.44, 4176.29, 6565.74, and 5536.42 cm^2^ after 15, 30, 45, 60, 75, and 90 days, respectively. Further, the average increase rate in the total leaf area between two consecutive observation time points was 259.04%, 303.84%, 280.55%, 57.21%, and −15.68%, respectively. It indicated that the total leaf area in all treatment combinations increased slowly in the seeding stage. After the jointing stage, the total leaf area increased rapidly and peaked after 75 days. After 90 days of sowing, the total leaf area decreased because the maize plants and leaves became senile.

**Figure 5:**
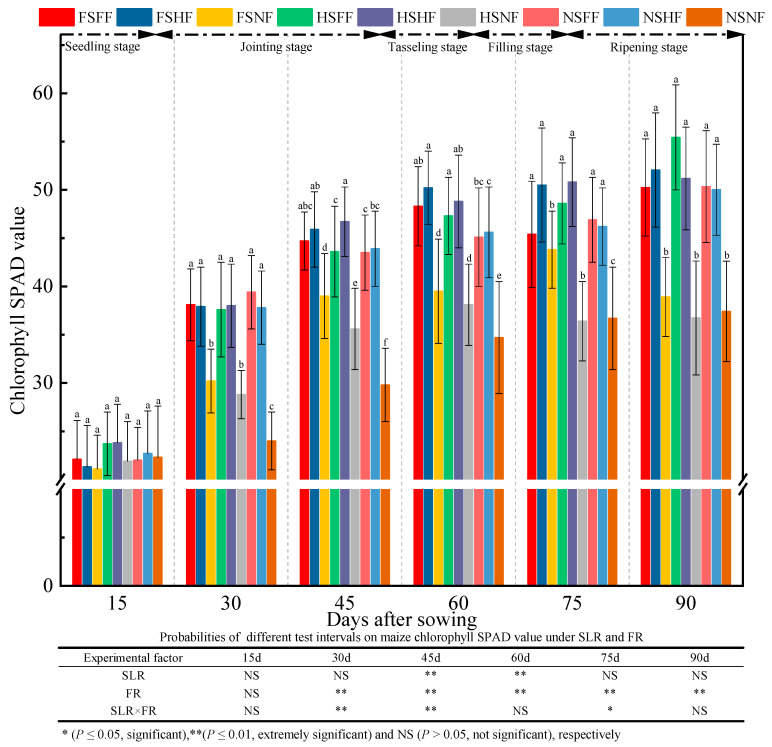
Effects of SLR and FR on the chlorophyll SPAD value. Note: Bars represent means ± standard deviation (*n* = 3) with different letters indicating significant differences based on LSD (*p* ≤ 0.05). SLR, sugarcane leaf return; FR, fertilizer reduction.

The SPAD (Soil and Plant Analyzer Development) value in all nine treatment combinations during the whole growth stage was as shown in Figure 5. A significant difference in SPAD value was not observed among nine treatment combinations in the seedling stage. The SPAD value was significantly higher (*p* < 0.05) under the FF and HF conditions than that under the NF condition. Under the NF condition, the SPAD value of the three SLR amounts was in the order FS > HS > NS. The statistical results of nine treatment showed that FR (except seedling stage) affected SPAD value significantly (*p* < 0.01). SLR (after 45 and 60 days of sowing) and combined treatment with SLR and FR were found to affect the SPAD value significantly (*p* < 0.05) after 30, 45, and 75 days. The increase rate in SPAD value between two consecutive observation time points was 55.2%, 19.5%, 6.7%, 1.9%, and 4.2%, respectively. The SPAD value increased slowly in the seedling stage. After the jointing stage, the SPAD value increased rapidly. After 90 days of sowing, the SPAD value was found to be stable at a certain level.

### 2.2. Maize Yield Component Factors and Yield

As depicted in Table 1, the analysis results of experimental treatments on maize yield component factors and yield showed that SLR, FR, and SLR and FR combined treatment significantly affected the ear diameter and plant height (*p* < 0.05). Ten maize yield component factors and maize yield were affected significantly by FR (*p* < 0.05), besides grain yield rate and HI. A significant difference in ear height and HI was not observed among nine treatment combinations. The reanalysis results of the effect of different experimental treatments of maize yield component factors and yield were as shown in Table 2. The effect of experimental treatment on four yield component factors (1000 kernel weight, number of productive ears, ear length, and plant air-dried weight) was at the maximum in the HSFF group. The effect of nine experimental treatments on ear diameter, rows per ear, ear height, and grain yield rate was the highest in the HSHF group. The effect of nine experimental treatments on ear weight per plant and maize yield was at the maximum in FSHF treatment. The HI had the maximum value in the FSNF. Under the NS condition, the increase rate of seven maize yield component factors (besides ear height, rows per ear, ear length, and grain yield rate) and maize yield were higher in FF/NF than that in HF/NF. The growth rate was obviously lower in FF/NF with FS and HS than in FF/NF without SLR (NS) in terms of seven maize yield component factors (ear weight per plant, number of productive ears, plant height, ear diameter, ear height, HI, and maize yield). Further, the increase rate was obviously lower in HF/NF with FS and HS than that in HF/NF without SLR (NS) in terms of five maize yield component factors (1000 kernel weight, plant height, ear diameter, ear height, and maize yield). The multiple comparison results indicated that the increase rate in FF/NF and HF/NF positively increased in terms of 10 maize yield component factors (except HI) and maize yield, yet the increase rate in FS/NS and HS/NS positively increased in terms of six yield component factors (ear weight per plant, number of productive ears, plant height, ear diameter, grain yield rate, and HI). However, the increase rate was obviously higher in FF/NF and HF/NF than that in FS/NS and HS/NS.

**Figure 6:**
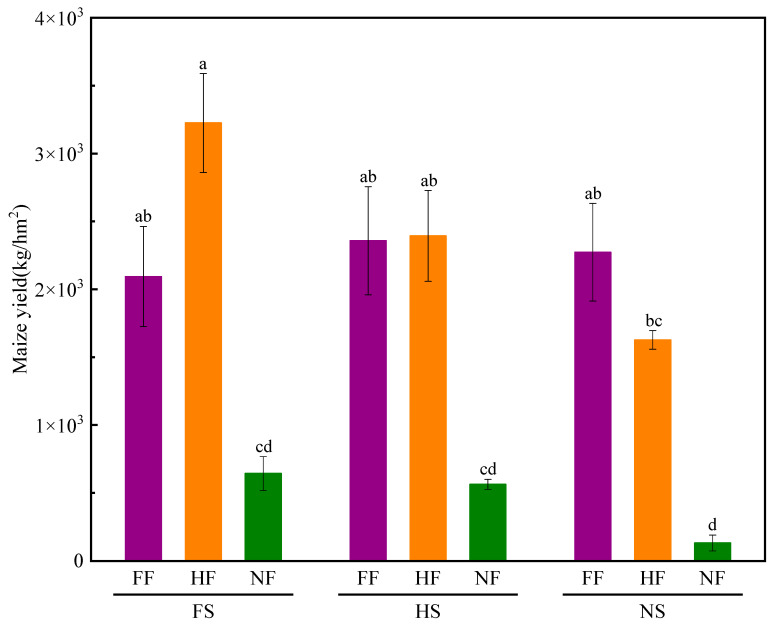
Effects of SLR and FR on maize yield. Note: Bars represent means ± standard deviation (*n* = 3) with different letters indicating significant differences based on LSD (*p* ≤ 0.05). SLR, sugarcane leaf return; FR, fertilizer reduction.

As shown in Figure 6, FR and combined treatment with SLR and FR significantly affected the maize yield (*p* < 0.05). Although SLR could increase maize yield, it was not significant. Compared with NSNF (CK), the maize yield in the FSFF, FSHF, FSNF, HSFF, HSHF, HSNF, NSFF, and HFNS treatments was higher by 1473.42%, 2323.96%, 383.57%, 1671.73%, 1698.76 %. 323.22%, 1608.33%, and 1122.90%, respectively. The maize yield of FSHF was 3225.08 kg/km^2^, which was the highest maize yield among the nine treatment combinations. Under the FS and HS conditions, the relative increase rate of FF/NF and HF/NF was 225.38% and 401.26%, and 318.63% and 325.01%, respectively. Meanwhile, under the NS condition, the relative increase rate of FF/NF and HF/NF was 1608.33% and 1122.90%, respectively. The maize yield was higher by 383.57% and 323.22% in FSNF and HSNF than that in NSNF. The experimental results indicated that SLR treatment alone could enhance maize yield, and the maize yield increased with FS and HS was higher than that with NS. The effect of SLR and FR combined treatment on maize yield was higher than that of FR and SLR application alone.

### 2.3. Soil Properties

**Figure 7:**
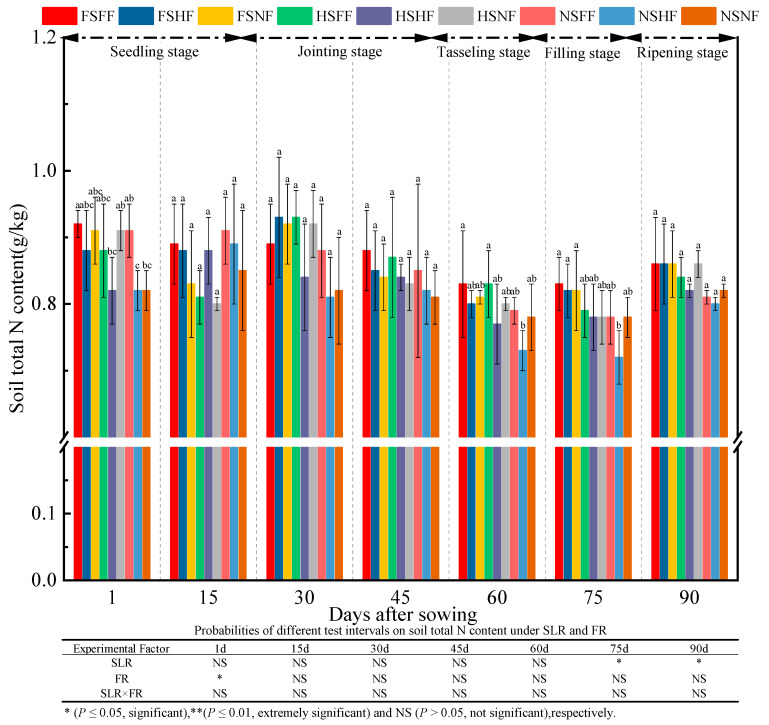
Effects of SLR and FR on soil TN content. Note: Bars represent means ± standard deviation (*n* = 3) with different letters indicating significant differences based on LSD (*p* ≤ 0.05). SLR, sugarcane leaf return; FR, fertilizer reduction.

The results of soil TN content at the seven test times are shown in Figure 7. Treatments with SLR (except 75 and 90 days), FR (besides 15 days), and SLR and FR combined had no significant effect on TN. The experimental factor on TN was marginal at seven observation time points, and the significant difference in TN among nine treatments was found merely after 1, 60, and 75 days. The average increase rate of TN between two consecutive observation time points was −1.7%, 2.6%, −4.4%, −5.9%, −0.6%, and 6.1%. Overall, TN content in all treatments initially varied and then leveled off at 0.80–0.86 g/kg at the end of the growth period. TN content in FSFF was always the highest in terms of the experimental factors at all observation time points (except 15 and 30 days). Irrespective of the kind of FR application, average TN content in three SLR amounts at seven observation time points (except 15 days) was in the order FS > HS > NS. Further, irrespective of the SLR content, TN content under three FR levels was in the order FF > HF > NF. Under FR condition, the increase in the rate of TN in FF/HF under FS and HS condition was lower than that under NS condition, possibly because of the consumption of soil N by soil microbes and the decomposition of sugarcane leaves.

**Figure 8:**
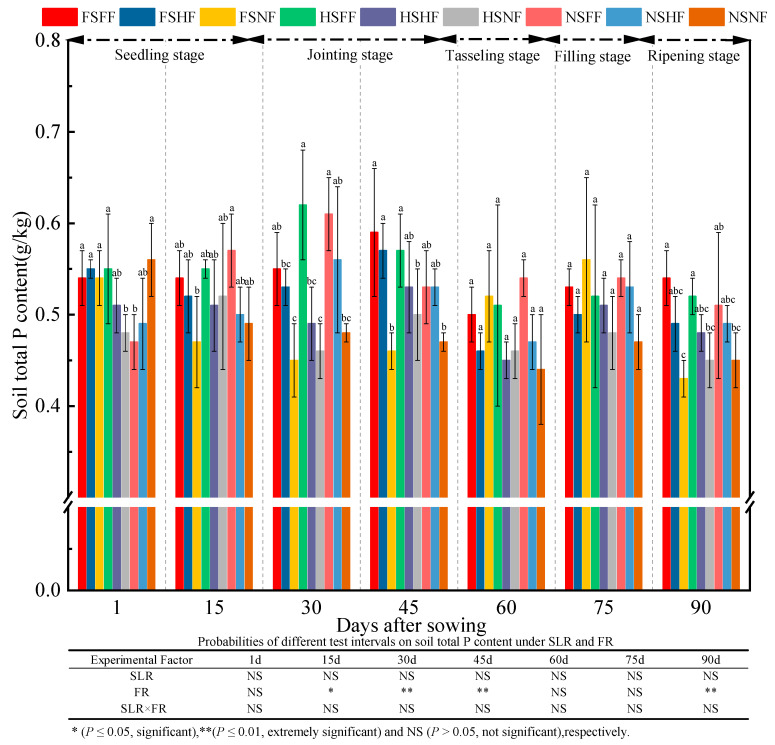
Effects of SLR and FR on soil TP content. Note: Bars represent means ± standard deviation (*n* = 3) with different letters indicating significant differences based on LSD (*p* ≤ 0.05). SLR, sugarcane leaf return; FR, fertilizer reduction.

Soil TP content in all treatment combinations during the whole experimental period was as shown in Figure 8. The statistical results of experimental factors on TP indicated that FR (in the jointing and tasseling stages) had significant effects on TP (*p* < 0.05) (Figure 8). Average TP content was 0.52, 0.52, 0.53, 0.53, 0.48, 0.52, and 0.48 g/kg at seven observation time points, respectively. The average increase rate of TP between two consecutive observation time points was −0.4%, 1.7%, 0%, −8.4%, 6.7%, and −6.0%, respectively, tested in seven time points. TP content after 1 day was higher by 7.0% than that after 90 days. TP content fluctuated during the whole experimental period. Overall, TP content leveled off at 0.45–0.54 g/kg at the end of the experiment, and the change trend on TP content was similar to that on TN content. In terms of experimental factors, the increase rate of TP under the FS and HS conditions was higher than that under the NS condition after 1, 45, 60, and 90 days. Meanwhile, irrespective of SLR treatment, TP content under three FR levels was in the order FF > HF > NF after 30, 45, 75, and 90 days.

**Figure 9:**
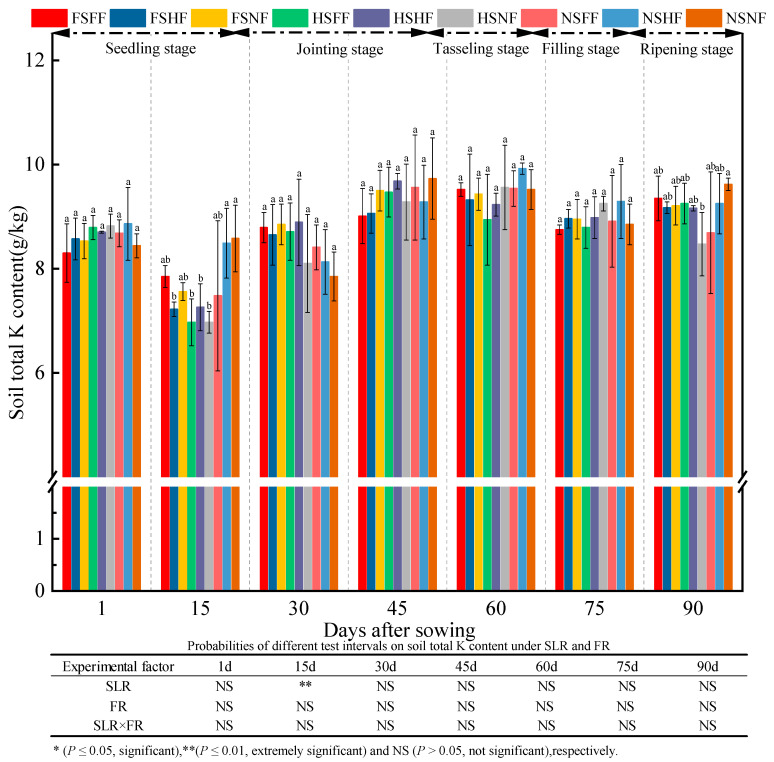
Effects of SLR and FR on soil TK content. Note: Bars represent means ± standard deviation (*n* = 3) with different letters indicating significant differences based on LSD (*p* ≤ 0.05). SLR, sugarcane leaf return; FR, fertilizer reduction.

As shown in Figure 9, statistical results of experimental treatments on soil TK content indicated that SLR (except 15 days), FR, and SLR and FR combined treatments had no significant influence on soil TK in general. At seven observation time points, the average TK content was 8.63, 7.60, 8.49, 9.40, 9.44, 8.97, and 9.13 g/kg, receptively. The average relative increase rate of TK between the two observation time points was −12.0%, 11.7%, 10.7%, 0.5%, −5.0%, and 1.8%, respectively. TK content fluctuated with the progress of the experiment. From 1 to 15 days, TK content began to decline and then increased gradually and peaked in the jointing stage. After 45 days, TK content had decreased to a certain extent. This might be because of the consumption of soil potassium by soil microbes and the decomposition of sugarcane leaves. For the decomposition of sugarcane leaves, the soil K nutrient was revealed from sugarcane leaves to the soil, resulting in a gradual increase in TK. At the end of the experiment, the average TK content was higher by 5.8% after 90 days than that after 1 day. In terms of experimental factors, the average increase rate on FF or HF vs. NF with SLR was higher than that without SLR after 15, 45, and 90 days, and the average increase rate of TK in FF and HF was higher than that in NS. The average increase rate of TK between FF/NF and HF/NF was not significant.

**Figure 10:**
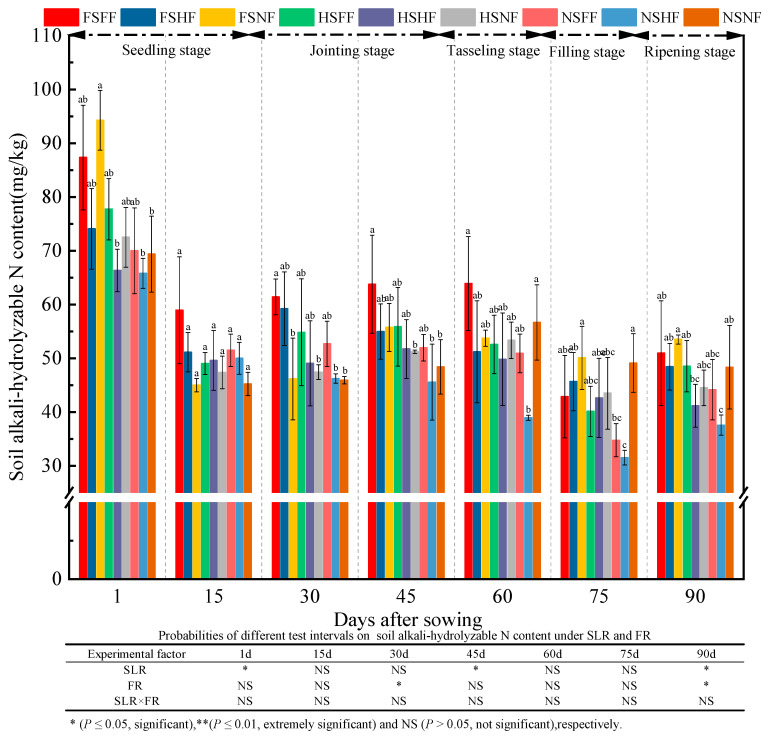
Effects of SLR and FR on soil alkali–hydrolysable N content. Note: Bars represent means ± standard deviation (*n* = 3) with different letters indicating significant differences based on LSD (*p* ≤ 0.05). SLR, sugarcane leaf return; FR, fertilizer reduction.

The assay results of soil AN content for all experimental treatments during the whole experimental stage were as shown in Figure 10. The average AN content was 75.27, 49.75, 51.44, 53.25, 52.34, 42.25, and 46.35 mg/kg after 1, 15, 30, 45, 60, 75, and 90 days, respectively. Further, the average relative increase rate of AN between two consecutive observation time points was −33.90%, 3.40%, 3.52%, −1.71%, −19.27%, and 9.70%, respectively. AN content in nine treatments was the highest after 1 day compared with other observation time points, and then declined rapidly after 15 days. From the jointing to the tasseling stage, AN content increased gradually among nine treatments. After 75 days, AN content in nine treatments decreased slightly and then increased to a certain extent in the ripening stage. The descending trend of AN content was due to the increased uptake of AN in soil with maize growth. Compared with AN content after 90 days, AN content on 1 day was 38.42% higher. Overall, AN content in nine treatments decreased gradually. Under the FS and HS conditions, the average increase rate of AN content in FF and HF compared with NF (CK) was 7.52%, 13.18%, 20.18%, 5.77%, 1.56%, −8.23%, and −0.317%, respectively. Under the NS condition, the average increase rate of AN content in FF and HF vs. NF was −2.12%, 12.17%, 7.66%, 0.76%, −20.74%, and −15.44%, respectively. The statistical analysis results suggested that SLR and FR had a significant effect on AN (*p* < 0.05) after 1, 45, and 90 days and after 45 and 90 days (*p* < 0.05), respectively.

**Figure 11:**
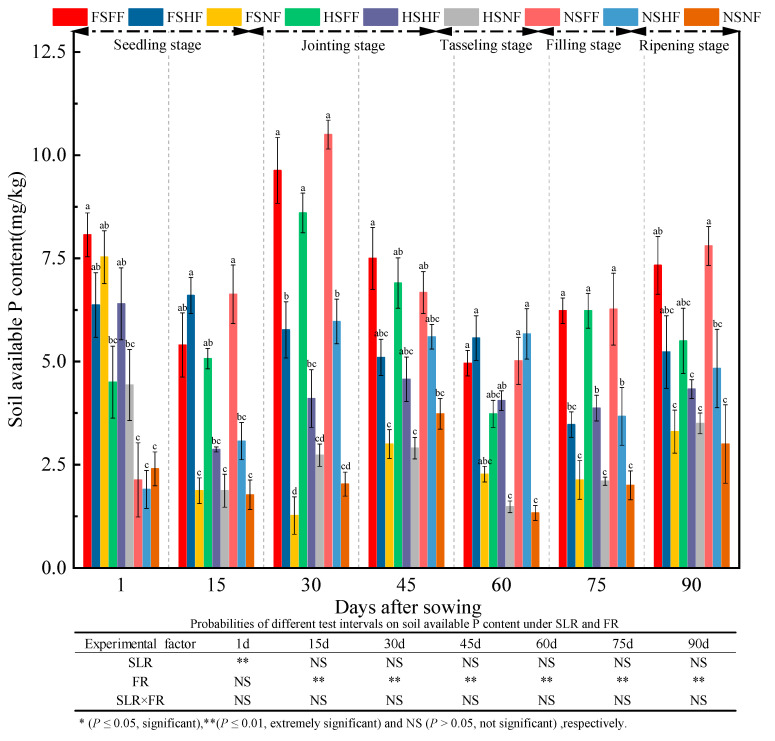
Effects of SLR and FR on soil AP content. Note: Bars represent means ± standard deviation (*n* = 3) with different letters indicating significant differences based on LSD (*p* ≤ 0.05). SLR, sugarcane leaf return; FR, fertilizer reduction.

The average AP content during the whole experimental period was 4.86, 3.91, 5.62, 5.11, 3.79, 4.00, and 4.98 mg/kg, respectively (Figure 11). Further, the average increase rate of AP content between two consequent observation time points was −19.6%, 44.0%, −9.2%, −25.9%, 5.5%, and 24.6%, respectively. The AP content on day 1 was lower by 2.5% compared with the AP content after 90 days. Overall, the same trend of AP content was observed in FS, HS, and NS treatments, which was in the order FH > HF > NF, and it was more obvious after 30, 45, 75, and 90 days. Under the FS and HS conditions, the average increase rate of AP content under FF and HF treatments vs. NF treatment was −9.45%, 166.58%, 319.45%, 103.88%, 147.39%, 134.09%, and 65.37%, respectively. Further, under NS condition, the average relative rate of AP content increase on FF and HF vs. NF was −16.04%, 174.01%, 305.67%, 64.48%, 301.88%, 148.50%, and 110.50%, respectively. After 1, 45, and 60 days, the average increase rate of AP content with SLR was always higher than that without SLR. The statistical results of the effects of experimental factors on the AP content indicated that FR had a significant effect on the AP content (*p* < 0.05) in the whole growth stage. SLR and SLR and FR combined treatment had almost no significant effects on soil AP.

**Figure 12:**
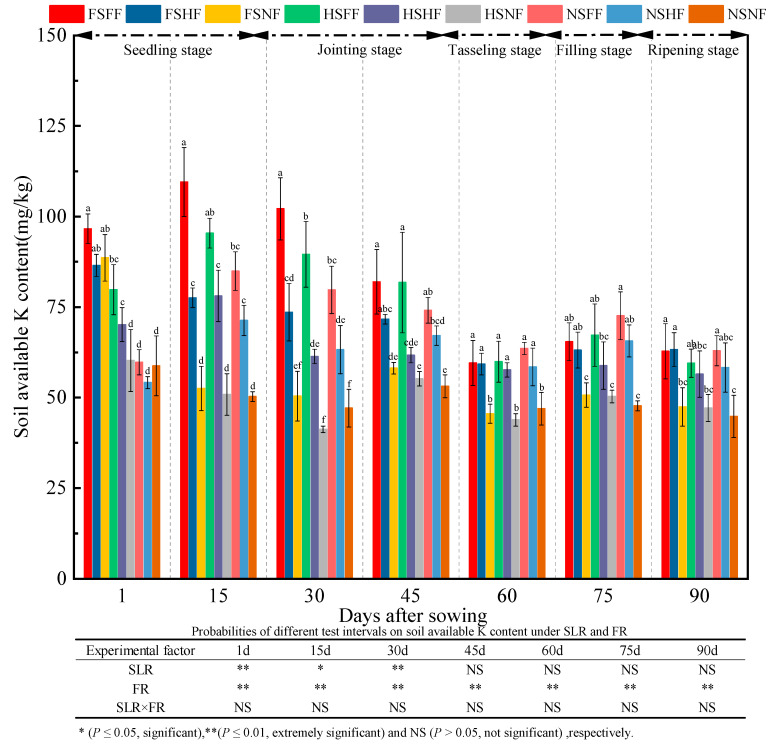
Effects of SLR and FR on the soil AK content. Note: Bars represent means ± standard deviation (*n* = 3) with different letters indicating significant differences based on LSD (*p* ≤ 0.05). SLR, sugarcane leaf return; FR, fertilizer reduction.

Soil AK content in all treatment combinations during the whole experimental period was as shown in Figure 12. The average AK content was 72.72, 74.48, 67.58, 67.19, 54.96, 60.16, and 55.83 mg/kg at seven observation time points, respectively. Further, the average increase rate of AK content between two consequent observation time points was 2.4%, −9.3%, −0.6%, −18.2%, 9.5%, and 23.2%, respectively. The AK content after 1 day was lower by 2.5% compared with the AK content after 90 days. Overall, the AK content decreased gradually. Under FS and HS condition, the AK content of three FR levels was in the order of FH > HF > NF, and it was more obvious after 15, 30, and 45 days. Under FS and HS conditions, the average relative increase rate of the AK content on FF and HF vs. NF was 13.90%, 74.39%, 78.65%, 31.07%, 32.26%, 26.05%, and 27.93%, respectively. Meanwhile, under the NS condition, the average relative increase rate of AP content on FF and HF vs. NF was −3.13%, 55.39%, 51.73%, 32.88%, 30.02%, 44.77%, and 35.23%, respectively. The average increase rate of AK content with SLR was higher than that without SLR under FF and HF after 1, 15, 30, and 60 days. The effects of experimental treatments on soil AK indicated that FR had an extremely significant effect on soil AK (*p* < 0.01). SLR had a significant effect on AK content (*p* < 0.05) after 1, 15, and 30 days. No significant interaction effect of SLR and FR was observed on soil AK.

**Figure 13:**
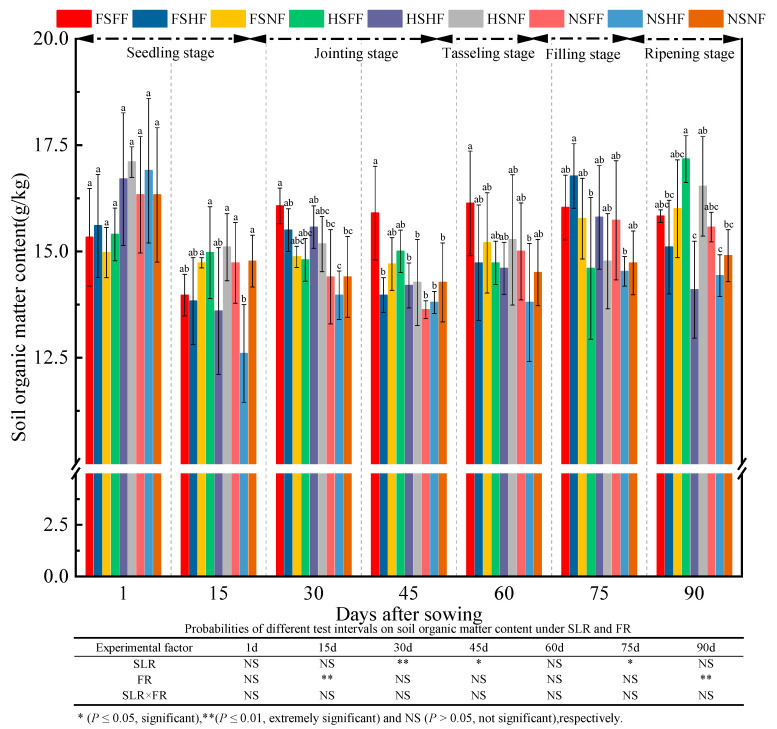
Effects of SLR and FR on the SOM content. Note: Bars represent means ± standard deviation (*n* = 3) with different letters indicating significant differences based on LSD (*p* ≤ 0.05). SLR, sugarcane leaf return; FR, fertilizer reduction.

The effect of experimental factors on SOM content indicated that SLR and FR had a significant effect on SOM content (*p* < 0.05) after 30, 45, and 75 days and after 15 and 90 days, respectively (Figure 13). However, the interaction effect of combined treatment with SLR and FR on SOM was not observed. The average SOM content was 16.07, 14.26, 14.97, 14.42, 14.88, 15.41, and 15.51 g/kg after 1, 15, 30, 45, 60, 75, and 90 days, respectively. Further, the average relative increase rate of the SOM content between two consecutive observation time points was −11.3%, 5.0%, −3.7%, 3.3%, 3.6%, and 0.6%, respectively. The SOM content on day 1 was higher by 3.5% compared with the SOM content after 90 days. Overall, the change trend of the SOM content decreased gradually. Under the FS and HS conditions, the SOM content of three FR levels was in the order of FF > HF > NF, and it was more obvious after the jointing and tasseling stages. Under FS or HS condition, the average relative increase rate of the SOM content on FF and HF vs. NF was −1.42%, −5.52%, 3.13%, 1.96%, −1.22%, 3.45%, and −4.38%, respectively. Meanwhile, under NS condition, the average relative increase rate of the SOM content on FF and HF vs. NF was 1.75%, −7.48%, −1.49%, −3.89%, −0.69%, 2.72%, and 0.67%, respectively. After 15, 30, 45, and 75 days, the average increase rate of the SOM content with SLR was higher than that without SLR.

**Figure 14:**
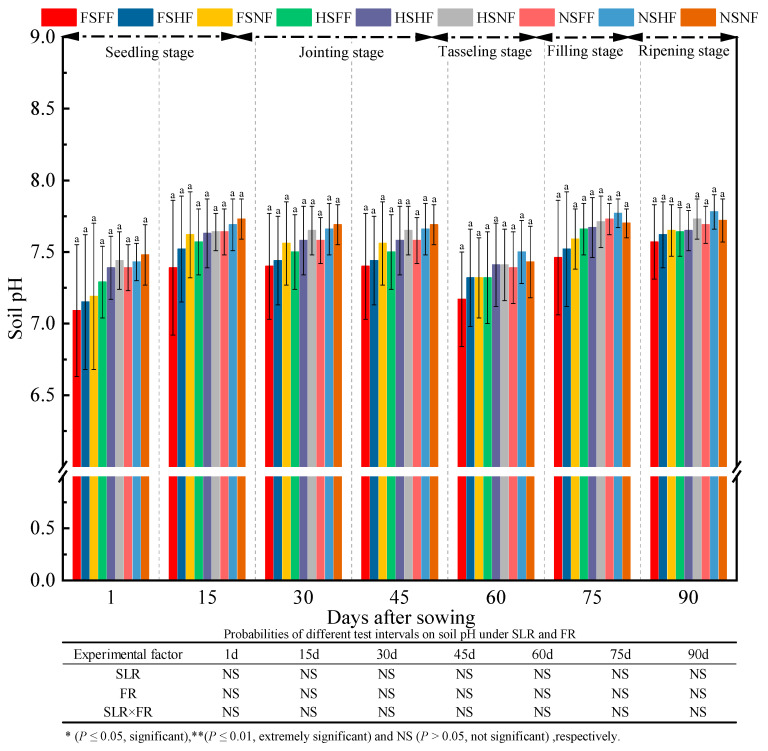
Effects of SLR and FR on the soil pH. Note: Bars represent means ± standard deviation (*n* = 3) with different letters indicating significant differences based on LSD (*p* ≤ 0.05). SLR, sugarcane leaf return; FR, fertilizer reduction.

The soil pH during the whole experimental period was as shown in Figure 14. The effect of experimental factors on soil pH showed that SLR, FR, and SLR and FR combined treatments had no significant effect on soil pH (*p >* 0.05). No significant difference in the soil pH was observed in nine treatment combinations at all observation time points. The average soil pH was 7.32, 7.60, 7.56, 7.56, 7.36, 7.65, and 7.67 after 1, 15, 30, 45, 60, 75, and 90 days, respectively. Further, the average relative increase rate of soil pH between two consequent observation time points was 3.9%, −0.5%, 0.0%, −2.6%, 3.8%, and 0.3%, respectively. The soil pH after 1 day was lower by 4.9% than that after 90 days. Overall, the soil pH increased slightly during the whole maize growth period. Under the FS and HS conditions, the soil pH of the three FR levels was in the order of NF > HF > FH; under the FS and HS conditions, the average relative increase rate of soil pH on FF and HF vs. NF was −1.16%, −1.34%, −1.64%, −1.64%, −0.82%, −0.95%, and −0.91% at seven observation time points, respectively. On the contrary, the average relative increase rate of the soil pH on FF and HF vs. NF was −0.94%, −0.84%, −0.91%, −0.91%, 0.20%, 0.65%, and 0.19% under the NS condition at all observation time points, respectively. The average increase in soil pH with SLR was lower than that without SLR.

**Figure 15:**
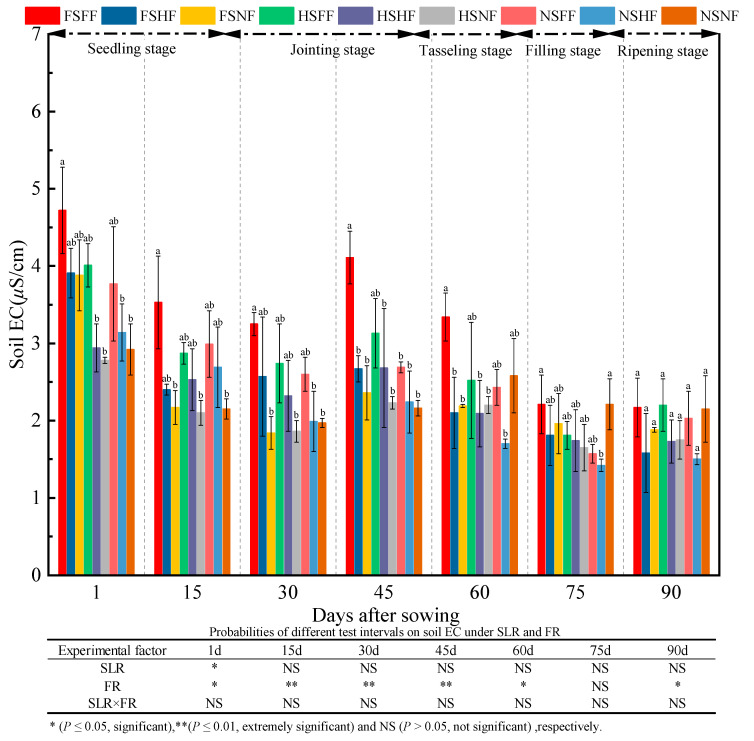
Effects of SLR and FR on soil EC. Note: Bars represent means ± standard deviation (*n* = 3) with different letters indicating significant differences based on LSD (*p* ≤ 0.05). SLR, sugarcane leaf return; FR, fertilizer reduction.

As shown in Figure 15, the effect on soil EC indicated that FR had a significant effect on soil EC during the whole experimental period (*p* < 0.05) except after 75 days. SLR (except day 1) and SLR and FR combined treatments had no significant effect on soil EC (*p <* 0.05). The average soil EC was 3.56, 2.60, 2.35, 2.70, 2.35, 1.82, and 1.89 μS/cm after 1, 15, 30, 45, 60, 75, and 90 days, respectively. Further, the average relative increase rate of soil EC between two consequent observation time points was −26.9%, −9.8%, 14.8%, −12.9%, −22.6%, and 3.7%, respectively. Compared with soil EC on day 1, soil EC after 90 days had decreased by 47.0%. A significant difference in soil EC in nine treatments was observed after 0–75 days. Overall, soil EC decreased gradually, and the significant difference in soil EC among nine treatment combinations almost disappeared at the end of the experiment. Under FS and HS condition, soil EC of the three FR levels was always as follows: NF > HF > FH after 1, 15, 30, and 45 days. Under the FS and HS conditions, the average relative increase rate of soil EC on FF and HF vs. NF was 18.11%, 32.60%, 47.09%, 36.96%, 14.49%, 5.06%, and 6.01% at all observation time points, respectively. Under the NS condition, the average relative increase rate of soil EC on FF and HF vs. NF was 18.32%, −0.84%, 32.09%, 16.50%, 14.12%, −19.96%, and −32.35% at the seven observation time points, respectively. The average increase rate of soil EC with SLR was always lower than that without SLR.

### 2.4. Correlation Analysis between Maize Growth and Soil Properties

The correlation coefficients of maize growth and soil properties were evaluated via the principal correlation analysis (Table 3). The pH was significantly positively correlated with SPAD, but negatively correlated with plant height. Significantly positive correlations were observed between plant height, stalk diameter, and number of fully developed maize leaves, and TK, AP, and SOM. The stalk diameter and number of fully developed maize plant leaves were significantly negatively correlated with EC. TN was significantly negatively correlated with plant height, stalk diameter, and number of fully developed maize plant leaves, but positively correlated with total leaf area. The correlation pattern of AK was similar to TN.

## 3. Discussion

### 3.1. Impacts of SLR and FR on Maize Growth

Dynamic changes in maize growth were important in evaluating SLR and FR performances. In the seedling stage, the changes in all growth indexes were nearly identical. Compared with the rest of the growth stages, maize growth rate was relatively slower in the seedling stage and the differences between experimental treatments were not obvious. Crop straw is a plentiful and inexpensive source of organic material, which usually affects crop growth [42,43,44]. Wei et al. [45] reported that rice straw return inhibited rice plant height, number of tillers, and biomass in the seedling stage, while it clearly improved rice growth after the jointing stage. Sun [46] and Fang [47] concurred with this result. In this study, plant height, stalk diameter, number of fully developed maize plant leaves, total leaf area, and SPAD value of FS were lower than those of HS after 30 days under the NF condition. However, this phenomenon disappeared after the jointing stage. The results of this study were similar to those of previous studies. These results implied that the available nutrients of sugarcane leaf residues for maize growth were needed for a certain period. Generally, the decomposition of sugarcane leaf requires nutrients from soil; as a result, the maize growth is suppressed in the maize seedling stage due to the high C/N ratio of sugarcane leaf residue and competition for soil N nutrients with sugarcane leaf decomposition. More availability of nutrients from sugarcane leaf would accelerate maize growth as time advances. In the present study, irrespective of the FF, HF, or NF conditions, the all-maize growth index was invariably higher under the FS and HS conditions than that under the NS condition after the seedling stage. This suggested that SLR significantly improved the maize growth rate more than inhibiting it during the whole maize growth period. We observed that the SLR’s effect irrespective of FS or HS without fertilization on maize growth was significantly lower than that of FS and HS with fertilization. Either SLR or fertilization could promote maize growth. Maize growth was inhibited when the plants were treated with FS without fertilization. The results of this study were consistent with the findings of Geng [48]. Fan [44] found that the effect of maize straw return was better on maize root growth than that of potassium fertilizer. In the present study, significant effects were observed with FR in terms of plant height, stalk diameter, number of five maize growth indexes. After the maize seedling stage, the effect of FF and HF on maize growth was better than that of NF under the FS and HS conditions. Meanwhile, the effect of HF was slightly lower than that of FF. The effect of FR was better on maize growth than that of SLR. This result was different from the findings of Fan [44]. Thus, SLR with reasonable fertilization would be the main driving factor for enhanced maize growth in SLR-treated fields.

Han [49] reported that the combined application of chemical and organic fertilizers could increase maize fresh and dry weights significantly. In this study, two-way ANOVA revealed that the effects of SLR or FR were significant on maize growth, while no significant interaction effects were observed on the most growth indexes (Figure 2, Figure 3, Figure 4, Figure 5, Figure 6 and Figure 7). The effect of HSFF treatment on five maize growth indexes was higher than that of other treatments. These results inferred that HS with FF application would be an optimized treatment combination in pot experimental conditions.

### 3.2. Impacts of SLR and FR on Maize Yield Component Factors and Yield 

Crop yield component factor and yield are good measurement indexes to examine the experimental treatment performance [50,51]. Previous studies showed that a combination of straw return and fertilizer was effective in enhancing crop yield [9,24,52]. Fan et al. [44] argued that the effect of potassium fertilizer application on maize grain yield was better than that of straw return alone. As observed in this study, either SLR or fertilization could increase maize yield. The input of sugarcane leaves would reduce chemical fertilizers consumption to some extent, compared with fertilizer application alone in red soil. However, the maize yield and yield component factors in SLR condition were similar to those under FF and HF (Table 2 and Table 3). These results indicated that SLR could compensate for the negative effects of FR input on maize yield, but the effect of SLR on maize yield components and yield was lower than that of FR, and hence SLR could not replace fertilization completely. Previous studies showed that straw return alone resulted in grain yield similar to NF in the long term [23], which was consistent with the results of this study. The highest yields were obtained in the FSHF, followed by the HSHF, HSFF, and NSFF. Combined treatment SLR and FR increased maize grain yield significantly rather than SLR application alone, which might be because of the slow release of nutrients and low nutrient content from sugarcane leaf [53]. In this study, the maize yield increased with SLR, and SLR and FR combined treatment significantly increased the grain yield than optimal chemical fertilization alone. We recommend FSHF and HSHF treatments to manage sugarcane leaf residues and FR in maize production.

### 3.3. Impacts of SLR and FR on Soil Properties 

Chen et al. [52] reported that straw return with chemical fertilizers did not increase TN content, but significantly enhanced the AP and AK contents during the long-term experiment. Liu et al. [24] indicated that TN and AN content were enhanced by wheat straw incorporation and nitrogen application. In this study, TN content in nine treatments was basically unchanged at most observation time points. The results showed neither SLR nor FR altered TN content significantly throughout the observation period. The results of this study were consistent with the findings of Chen [52]. Therefore, a small amount of SLR and fertilizer could not affect the TN in a relatively short period. 

Previous studies [54,55,56] suggested that straw inputs might not always increase soil AP content. Li et al. [57] reported that straw return and earthworm inoculation could reduce soil potassium absorption efficiency. However, Pu et al. [54] reported that maize straw input increased soil potassium transformation except for soil AP. The results of this study indicated that although no significant effect of SLR was observed on soil TP content, the TP and AP content were in the order of FS > HS > NS.

Study [24] showed that the combination of straw return and nitrogen fertilizer was an effective strategy for increasing soil AN content. Study [55] demonstrated that mineral phosphorus fertilizer and maize straw incorporation did not decrease soil labile inorganic potassium concentration. The study by Fan et al. [44] indicated that the impact on soil potassium availability of the interaction of straw return and the potassium fertilizer application was better than the effect of straw return or the application of potassium fertilizer alone. In this study, the interaction effect of SLR and FR on soil TK content was not found. The conclusions of this study were not consistent with those of previous studies. It might be due to the lower SLR and fertilization amount and a short test cycle. The contribution of crop residues to soil properties may be significant only under large amounts of residue return and may reach a higher available concentration to the soil. The high C/N ratio of sugarcane leaf residues results in significant nitrogen immobilization due to increased soil microbiological activity caused by energy input into the soil, leading to slower rates of nitrogen release in the short term. In this study, SLR could increase soil nutrient availability, and the effect of SLR was lower than that of FR. This may result in an increased crop nitrogen requirement and lowered sugarcane root sprouting and yield, because sugarcane leaves have a high C/N ratio (80–110:1) [20,58].

The correlational studies [59,60,61] claimed that the straw return practice was essential to SOM accumulation. Guo et al. [62] found that wheat straw incorporation altered and improved SOM during 30 years of field experiments. Li et al. [63] indicated that urea fertilization increased SOM content by decreasing the decomposition of SOM and maize straw. In this study, different effects on the SOM content were observed in nine treatments s after 30, 45, and 60 days, and the SOM content was in the order of FS > HS > NS. Under the NS condition, the SOM content in FF and HF was usually higher than that of NF, except after 15 and 45 days. Some scholars did not agree with the opinion [64,65,66] that straw return could increase SOM content. The reason was that organic matter application in the soil could not affect the chemical properties and SOM quality. Straw return even supported the mineralization process of SOM and thus led to a decrease in the SOM content. These conflicting results might be due to the difference in soil texture, climate conditions, and amount of organic material input, among other factors. In this study, the change trend of the SOM content was inconsistent. It might be due to a smaller amount of SLR, and no significant interaction effect of the SLR and FR combined treatment. 

Zhang et al. [67] reported that cotton straw retention increased soil pH, whereas fertilization reduced it. Ran et al. [68] indicated that straw return significantly reduced soil pH. Chao et al. [62] argued that the long-term straw incorporation accentuated soil acidification. In this study, SLR and FR did not significantly affect soil pH. These contradictory findings might be due to the short-term pot experiments. Ran [68] and Song [69] reported that straw return reduced soil EC. In this study, irrespective of FF and HF or NF, EC in the three SLR levels was in the order of FS > HS > NS. Further, EC in the three FR levels was in the order FF > HF > NF. The aforementioned findings could be explained by the significant effect of FR on soil EC, but the effect of SLR was not significant. This was why the results of this study were inconsistent with previous findings. 

In this study, a significant interaction effect of SLR and FR on the soil properties index was not found. FR had a significant effect on the TP, AP, AK, and EC. The effect of SLR on soil properties was nominal due to low SLR and nutrient content, and hence the role of SLR on the soil properties was ignored. Positive correlations were observed between plant height, stalk diameter, and number of fully developed maize plant leaves with TK, AP, SOM. This also suggested to some extent that the increase in TK, AP, and SOM could enhance maize growth and yield.

## 4. Materials and Methods

### 4.1. Experimental Materials

The pot experiment was conducted in a plastic greenhouse (latitude: 23°12′42″ N, longitude: 108°11′07″ E, altitude: 145 m) in the Guangxi Vocational College of Water Resources and Electric Power, Nanning, Guangxi, China. The collected soil was red soil (Orthic Acrisol, Food and Agriculture Organization–United Nations Educational, Scientific and Cultural Organization system) with a soil texture of clay, derived from the experimental station of the Guangxi Vocational College of Water Resources and Electric Power. Before the test soil was used, it was air dried and crushed to pass through a 1.0-cm sieve and mixed thoroughly. The initial soil properties were as follows: pH, 7.02; EC, 80.9 ms/m; soil organic matter (SOM), 18.0 g/kg; total nitrogen (TN) content, 1.06 g/kg; total phosphorus (TP) content, 0.49 g/kg; total potassium (TK) content, 8.59 g/kg; alkali–hydrolyzable nitrogen (AN) content, 81.5 mg/kg; available phosphorus (AP) content, 2.0 mg/kg; and available potassium (AK) content, 52.0 mg/kg. The sugarcane leaves were obtained from the sugarcane experiment station of the Guangxi Academy of Agricultural Sciences, Nanning, Guangxi, China. Before the use of the sugarcane leaf residues, they were dried at 75℃ to a constant weight and artificially chopped to a length of 1.0–2.0 cm. The properties of the sugarcane leaves were as follows: TN, 5.56 g/kg; TP, 1.24 g/kg; TK, 4.74 g/kg; total organic carbon content, 557.52 g/kg; and C/N ratio, 98.24.

The hybrid maize (*Zea mays* L.) variety, with Yidan No. 629, was selected for this study. It is cultivated widely in South China. Maize was sown on 16 March 2022, with two seeds per bucket, 5.0 cm apart and 2.0 cm below the soil surface. All seeds sprouted completely on March 22. On March 25, one seedling was removed from each pot, and only the stronger seedling was retained. The maize was harvested on June 14. The whole growth period was 90 days from March 16 to June 14. The maize plants were irrigated artificially with the same weight running water every time. Manual weeding and pest control for uniformity were performed during the whole growth period.

### 4.2. Experimental Design

The pot experiment was set up using the randomized block design with SLR and FR as the experimental factors. Nine treatment combinations were established, using three different amounts of SLR (full SLR (FS), 120 g/pot; half SLR (HS), 60 g/pot; and no SLR (NS) with three FR levels including full fertilizer (FF), 4.50 g N/pot, 3.00 g P_2_O_5_/pot, and 4.50 g K_2_O/pot; half fertilizer (HF), 2.25 g N/pot, 1.50 g P_2_O_5_/pot, and 2.25 g K_2_O/pot; and NF), without nitrogen, phosphorous, and potassium added, with six replications. Maize was planted in black plastic buckets, with 30 kg of red soil added to each bucket on an air-dried weight basis. The SLR content was calculated as 9.0 t/hm^2^ using the average air-dried weight of sugarcane leaves in Guangxi [70,71], and the average surface soil weight was 2250 t/hm^2^ [72]. Urea (N, 46.0%), calcium superphosphate (P_2_O_5_, 12.0%), and potassium chloride (K_2_O, 60.0%) were used as nitrogen, phosphorous, and potassium fertilizers, respectively. All fertilizers were applied with the analytical reagent and mixed into the soil in powdered form at the beginning of the experiment, with no topdressing. The experimental design and the nutrient input of nine experimental treatments were followed as shown in Table 4.

### 4.3. Plant Measurements

#### 4.3.1. Maize Growth

Five parameters of maize growth, including plant height, stalk diameter, number of fully developed maize plant leaves, total leaf area, and chlorophyll SPAD reading value, were measured on 31 March, 15 April, 30 April, 15 May, 30 May, and 14 June 2022, respectively, with intervals of 15 days after the maize was sown (except on the first day). Before maize tasseling, the plant height of maize was measured from the ground to the highest point of the naturally extended leaves. After maize tasseling, the height was measured from the ground to the tip of the spike. The stalk diameter of maize was determined using a digital caliper (1501; AIRAJ Inc., Qingdao, China) 5 cm above the ground. The number of fully developed maize plant leaves were counted. For each fully developed maize plant leaf, the length and maximum width were measured using a flexible rule, and the leaf area was estimated using the following Equation (1) [73,74]:

Leaf area = leaf length × leaf width × 0.75(1)

The chlorophyll content of the maize plant was estimated using a Model SPAD-502 plus a hand-held chlorophyll meter (Konica Minolta Optics, Inc., Tokyo, Japan). For each sampled maize plant, the average SPAD value of all fully developed leaves was deemed as maize leaf chlorophyll content.

#### 4.3.2. Maize Yield Component Factors and Yield

All maize plants were harvested after 90 days of sowing, and then the details of the dry biomass accumulation, maize grain, straw, and root were recorded separately. The maize biomass and the grain yield of all plants selected from the same treatment were measured after 7 days of air drying. In this study, 10 maize yield component factors were used to evaluate the effects of SLR and FR on maize yield component factors. All yield component factors were analyzed statistically, including ear weight per plant, 1000 kernel weight, number of productive ears, rows per ear, ear diameter, ear length, plant air-dried weight, ear height, grain yield rate, and harvest index (HI). The grain yield rate was calculated for each pot using the following Equation (2):

(2) $$\mathrm{Grain}\;\mathrm{yield}\;\mathrm{rate}=\;\frac{\mathrm{air}-\mathrm{dried}\;\mathrm{kernel}\;\mathrm{weight}\;}{\mathrm{air}-\mathrm{dried}\;\mathrm{ear}\;\mathrm{weight}}\times 100\%\;$$

The HI was then calculated for each pot using the following Equation (3) [43]:

(3) $$\mathrm{HI}=\frac{\mathrm{air}-\mathrm{dried}\;\mathrm{grain}\;\mathrm{weight}\;}{\mathrm{air}-\mathrm{dried}\;\mathrm{biomass}\;\mathrm{weight}\;}\times 100\%\;$$

The maize yield from different treatments was calculated using the corresponding maize yield component factors.

### 4.4. Soil Sampling and Measurements

Soil sampling was carried out on 16 March, 31 March, 15 April, 30 April, 15 May, 30 May, and 14 June 2022, with an interval of 15 days after the maize was sown. Meanwhile, the maize growth in all nine treatment combinations was observed and recorded (except on day 1). Three soil cores were collected from each bucket at the same soil depths (0–10 cm). Fresh soil samples from each bucket were mixed thoroughly to form a composite sample. At every observation time point, 162 soil cores were collected and formed 54 composite soil samples. The soil samples from the same treatment were replicated six times. The assay results were based on the air-dried soil weight.

Soil pH and EC were measured using a soil:water ratio of 1:5 simultaneously. Soil pH was determined using a glass electrode (pHS-3C; Inesa Scientific Instrument Co., Ltd., Shanghai, China). Soil EC was measured using a conductivity meter (DDSJ-350; Inesa Scientific Instrument Co., Ltd., Shanghai, China). SOM was determined by wet oxidation using the acidified dichromate method. Soil TN content was measured using an automatic Kjeldahl nitrogen analyzer (8400; Foss Co., Ltd., Denmark) following the manufacturer’s protocols. Soil TP content was measured with an ultraviolet and visible spectrophotometer (T6; Puxi Co., Ltd., Beijing, China) using the molybdenum blue method. Soil TK content was measured using a flame photometer (FP640; Inesa Scientific Instrument Co., Ltd.). Soil alkali–hydrolyzable nitrogen (AN) content was determined using the diffusion absorption method. Soil AP content was extracted using 0.5 mol/L NaHCO_3_ solution and then measured using the molybdenum antimony-D-isoascorbic acid colorimetry method. Soil AK content was extracted using 1.0 mol/L NH_4_OAc and AK and TK content were determined using the same flame photometry technique.

### 4.5. Data Analysis

The maize growth, yield, and soil properties were calculated under the same SLR condition after FR treatments (FF and HF) compared with NF treatment. The relative increase rate for different treatments on maize growth, yield, and soil properties was calculated using the following Equation (4):

(4) $$\mathrm{Relative}\;\mathrm{increase}\;\mathrm{rate}\;(\%)=\frac{\mathrm{observation}\;\mathrm{value}\;\mathrm{of}\;\mathrm{FF}\;\mathrm{or}\;\mathrm{HF}\;-\;\mathrm{observation}\;\mathrm{value}\;\mathrm{of}\;\mathrm{NF}}{\mathrm{observation}\;\mathrm{value}\;\mathrm{of}\;\mathrm{NF}}\times 100\%\;$$

Multivariate analysis of variance (ANOVA) was performed following the general linear model univariate procedure using SPSS Statistics 27.0 software (IBM, NY, USA). The two-way ANOVA was performed for different SLR and FR levels to examine their impact on maize growth, yield, and soil properties. Significant differences between different treatments were calculated using the least significant difference (LSD) method (*p* < 0.05). The correlation between maize growth and soil proprieties was assessed via the two-tailed significance test using the Pearson coefficient.

## 5. Conclusions

Both SLR and FR could enhance maize growth, yield, and soil properties to some extent. In this study, the maize growth, yield, and soil properties were significantly affected by FR, but not by SLR. The interaction between FR and SLR could not influence maize growth, but ultimately affected maize yield. The effects of FR on maize growth, yield, and soil properties were higher than that of SLR. Plant height, stalk diameter, and number of fully developed maize plant leaves were positively correlated with soil TK, AP, and SOM. The experimental results indicated that the application of a reasonable amount of fertilizer combined with half SLR enhanced the growth and yield of maize and improved soil properties. The FSHF was found to be the optimal treatment combination in nine treatments. Future studies need to focus more on the SLR and FR combination to understand how SLR/FR–soil interactions affect below-ground ecological processes in agricultural ecosystems during long-term field conditions.

## Figures and Tables

**Table 1:** Effects of SLR and FR on maize yield component factors.

Experimental Treatment	Ear Weight per Plant (g)	1000 Kernel Weight (g)	Number of Productive Ears	Rows per Ear	Ear Diameter (mm)	Ear Length (cm)	Plant Air-Dried Weight (g)	Ear Height (cm)	Grain Yield Rate (%)	Harvest Index (%)
FSFF	198.87 ± 17.41 a	299.21 ± 16.43 ab	1.14 ± 0.18 ab	13.75 ± 1.67 ab	41.93 ± 6.29 a	18.86 ± 2.78 a	530.50 ± 58.45 ab	106.13 ± 18.58 a	54.36 ± 8.55 b	40.3 ± 5.7 a
FSHF	247.56 ± 17.45 a	281.57 ± 14.29 b	1.29 ± 0.29 a	14.00 ± 1.33 ab	40.75 ± 8.71 ab	18.26 ± 4.46 a	502.86 ± 62.26 ab	108.57 ± 12.07 a	53.32 ± 9.33 b	48.9 ± 7.9 a
FSNF	54.21 ± 7.94 cd	255.97 ± 22.33 b	0.71 ± 0.19 bc	12.00 ± 1.15 c	34.95 ± 8.52 bc	13.86 ± 2.70 b	162.86 ± 49.57 c	69.64 ± 14.47 b	47.30 ± 8.46 c	50.7 ± 8.9 a
HSFF	184.10 ± 17.34 a	383.62 ± 23.82 a	0.86 ± 0.18 abc	14.00 ± 2.53 ab	42.09 ± 7.05 a	19.93 ± 1.13 a	596.29 ± 28.85 a	108.07 ± 15.58 a	58.34 ± 9.66 ab	33.5 ± 2.9 a
HSHF	229.80 ± 15.05 a	292.69 ± 23.47 ab	1.14 ± 0.18 ab	14.50 ± 1.77 a	42.96 ± 6.50 a	19.81 ± 2.28 a	505.71 ± 25.07 ab	109.14 ± 8.17 a	66.75 ± 9.32 a	45.5 ± 5.5 a
HSNF	76.85 ± 6.82 bcd	251.19 ± 26.87 b	0.57 ± 0.13 c	12.57 ± 0.98 bc	38.14 ± 1.46 ab	12.79 ± 2.04 b	163.33 ± 34.45 c	72.86 ± 6.94 b	40.43 ± 7.35 cd	46.8 ± 9.4 a
NSFF	158.09 ± 12.14 ab	341.46 ± 21.52 ab	1.29 ± 0.19 a	14.00 ± 1.41 ab	41.54 ± 8.08 a	18.50 ± 2.14 a	560.00 ± 80.00 ab	104.14 ± 10.39 a	51.72 ± 7.21 b	36.0 ± 2.2 a
NSHF	146.94 ± 19.16 abc	304.43 ± 26.92 ab	1.00 ± 0.00 abc	14.25 ± 1.28 ab	40.56 ± 6.53 ab	18.93 ± 0.98 a	468.57 ± 66.19 b	107.64 ± 6.18 a	60.33 ± 6.09 ab	33.3 ± 5.9 a
NSNF	35.12 ± 6.83 d	247.30 ± 24.22 b	0.57 ± 0.13 c	12.57 ± 1.51 bc	29.49 ± 4.44 c	13.93 ± 3.89 b	120.00 ± 30.9 c	44.79 ± 12.03 c	40.96 ± 9.01 cd	35.5 ± 4.6 a
SLR	NS	NS	NS	NS	NS	NS	NS	<0.01	NS	NS
FR	<0.01	<0.01	<0.01	<0.01	<0.01	<0.01	<0.01	<0.01	NS	NS
SLR×FR	NS	NS	NS	NS	NS	NS	NS	<0.01	NS	NS

Note: Data (mean ± standard deviations, *n* = 3) with different letters indicate a statistically significant difference based on LSD (*p ≤* 0.05). The symbols in the following tables and figures are the same as those in this table.

**Table 2:** Reanalysis results of different experimental treatments of maize yield component factor and yield.

Yield Component Factor	Treatment Acronyms	Maximum Value	Unit	FF/NF (%)			HF/NF (%)			Multiple Comparison Results			
				FS	HS	NS	FS	HS	NS	FS/NS (%)	HS/NS (%)	FF/NF (%)	HF/NF (%)
Ear weight per plant	FSHF	247.56	g	266.85	139.56	350.14	356.67	199.02	318.39	40.10	47.90	285.77	211.6
1000 kernel weight	HSFF	383.62	g	16.89	52.72	38.08	10.00	16.52	23.10	−8.44	−0.05	34.73	15.51
Number of productive ears	HSFF	1.29	–	60.56	50.88	126.32	81.69	100.00	75.44	22.09	10.47	77.42	83.87
Rows per ear	HSHF	14.50	–	14.58	11.38	11.38	16.67	15.35	13.37	−2.24	0.35	14.94	12.37
Ear diameter	HSHF	43.96	mm	19.97	10.36	40.86	16.60	12.64	37.54	3.92	8.60	22.21	20.88
Ear length	HSFF	19.93	cm	36.08	55.82	32.81	31.75	54.89	35.59	−0.48	1.91	41.00	40.01
Plant air-dried weight	HSFF	596.29	G	225.74	265.08	366.67	208.77	209.62	290.48	−0.97	9.78	277.24	229.41
Ear height	HSHF	109.14	cm	52.40	48.33	132.51	55.90	49.79	140.32	10.16	13.06	69.98	73.72
Grain yield rate	HSHF	66.75	%	14.93	44.30	26.27	12.73	65.10	47.29	25.16	4.29	55.55	21.42
Harvest index	FSNF	50.7	%	−20.51	−28.42	1.41	−3.55	−2.78	−6.20	34.85	19.52	−21.76	−1.52
Maize yield	FSHF	3225.08	kg/hm^2^	225.38	318.63	1608.33	401.26	325.01	1122.90	−3.14	−29.42	941.32	695.04

**Table 3:** Correlation analysis between maize growth and soil properties.

Index	pH	EC	TN	TP	TK	AN	AP	AK	SOM
Plant height	−0.18 *	−ns	−0.24 **	ns	0.52 **	−ns	0.40 **	−ns	0.20 **
Stalk diameter	ns	−0.41 **	−0.34 **	ns	0.35 **	−0.39 **	0.21 **	−0.16 *	0.31 **
Number of fully developed maize plant leaves	−ns	−0.42 **	−0.36 **	ns	0.41 **	−0.36 **	0.25 **	−0.22 **	0.34 **
Total leaf area	ns	ns	0.21 **	ns	−0.47 **	ns	−ns	0.25 **	−0.18 *
SPAD	0.24 **	−ns	ns	−ns	−0.33 **	−ns	−0.43 **	−ns	−0.20 **

‘−’ and ‘ns’ represent negative correlation and not significant, respectively. * *p* < 0.05; ** *p* < 0.01.

**Table 4:** Experimental design and nutrient input of experimental treatments.

Treatment Combination Name	Treatment Acronyms	SLR Content per Pot (g)	FR Content per Pot (g) (Fertilization Type/Weight)	Nutrient Input (g)					
				SLR/pot			FR/pot		
				N	P_2_O_5_	K_2_O	N	P_2_O_5_	K_2_O
Full sugarcane leaf return + Full fertilizer	FSFF	120.0	CO(NH_2_)_2_/9.78 + Ca(H_2_PO_4_)_2_/25.00 + KCl/7.18	0.68	0.15	0.56	4.50	3.00	4.50
Full sugarcane leaf return + Half fertilizer	FSHF	120.0	CO(NH_2_)_2_/4.89 + Ca(H_2_PO_4_)_2_/12.50 + KCl/3.59	0.68	0.15	0.56	2.25	1.50	2.25
Full sugarcane leaf return + No fertilizer	FSNF	120.0	CO(NH_2_)_2_/0 + Ca(H_2_PO_4_)_2_/0 + KCl/0	0.68	0.15	0.56	0	0	0
Half sugarcane leaf return + Full fertilizer	HSFF	60.0	CO(NH_2_)_2_/9.78 + Ca(H_2_PO_4_)_2_/25.00 + KCl/7.18	0.34	0.07	0.28	4.50	3.00	4.50
Half sugarcane leaf return + Half fertilizer	HSHF	60.0	CO(NH_2_)_2_/4.89 + Ca(H_2_PO_4_)_2_/12.50 + KCl/3.59	0.34	0.07	0.28	2.25	1.50	2.25
Half sugarcane leaf return + No fertilizer	HSNF	60.0	CO(NH_2_)_2_/0 + Ca(H_2_PO_4_)_2_/0 + KCl/0	0.34	0.07	0.28	0	0	0
No sugarcane leaf return + Full fertilizer	NSFF	0	CO(NH_2_)_2_/9.78 + Ca(H_2_PO_4_)_2_/25.00 + KCl/7.18	0	0	0	4.50	3.00	4.50
No sugarcane leaf return + Half fertilizer	NSHF	0	CO(NH_2_)_2_/4.89 + Ca(H_2_PO_4_)_2_/12.50 + KCl/3.59	0	0	0	2.25	1.50	2.25
No sugarcane leaf return + No fertilizer (CK)	NSNF	0	CO(NH_2_)_2_/0 + Ca(H_2_PO_4_)_2_//0 + KCl/0	0	0	0	0	0	0

## Data Availability

All data included in the main text.

## References

[1] Chen D., Zhou W., Yang J., Ao J., Huang Y., Shen D., Jiang Y., Huang Z., Shen H. (2021). Effects of Seaweed Extracts on the Growth, Physiological Activity, Cane Yield and Sucrose Content of Sugarcane in China. Front. Plant Sci..

[2] Liu H., Yang J. (2022). Discussion on Mechanization Technology of Sugarcane Production in Guangxi. Int. J. Manag. Educ. Hum. Dev..

[3] Chen B., He Z., Liang Q., Tang X., Lin T., Liu Y. (2021). Comparative Analysis of Sugar Production Cost in Guangxi and World Major Producing Countries. Asian Agric. Res..

[4] Qi-Zhan T., Gui-Fen C., Zhong L., Cheng-Yan M., Ming-Hua G. (2009). Several sugarcane cultivars residues quantity and nutrient content of the residue. Soil Fertil..

[5] Li Y.M., Du Y.X., Zhou Y.S., Zhang S.Y. (2018). Research Progress of Sugarcane Leaves. Food Ind..

[6] Gullett B.K., Touati A., Huwe J., Hakk H. (2006). PCDD and PCDF Emissions from Simulated Sugarcane Field Burning. Environ. Sci. Technol..

[7] Razafimbelo T., Barthes B., Larré-Larrouy M., De Luca E.F., Laurent J., Cerri C.C., Feller C. (2006). Effect of sugarcane residue management (mulching versus burning) on organic matter in a clayey Oxisol from southern Brazil. Agric. Ecosyst. Environ..

[8] Dai H., Wang Y., Wen F., Tang Y. (2020). Analysis and Experiment of Flow Field in Sugarcane Leaf Cutting and Returning Machine. IOP Conf. Series Mater. Sci. Eng..

[9] Chen L., Sun S., Yao B., Peng Y., Gao C., Qin T., Zhou Y., Sun C., Quan W. (2022). Effects of straw return and straw biochar on soil properties and crop growth: A review. Front. Plant Sci..

[10] Sha Z., Ma X., Wang J., Lv T., Li Q., Misselbrook T., Liu X. (2020). Effect of N stabilizers on fertilizer-N fate in the soil-crop system: A meta-analysis. Agric. Ecosyst. Environ..

[11] Silva A.G.B., Lisboa I.P., Cherubin M.R., Cerri C.E.P. (2019). How Much Sugarcane Straw is Needed for Covering the Soil?. Bioenerg. Res..

[12] Galdos M., Cavalett O., Seabra J.E., Nogueira L.A.H., Bonomi A. (2013). Trends in global warming and human health impacts related to Brazilian sugarcane ethanol production considering black carbon emissions. Appl. Energy.

[13] Cerri C.C., Galdos M.V., Maia S.M.F., Bernoux M., Feigl B.J., Powlson D., Cerri C.E.P. (2011). Effect of sugarcane harvesting systems on soil carbon stocks in Brazil: An examination of existing data. Eur. J. Soil Sci..

[14] Leite L.F.C., Sagrilo E., de Araújo A.S.F., de Souza H.A. (2018). Short-term effect of sugarcane straw on soil organic carbon pools. Embrapa Meio-Norte-Artig. Periódico Indexado.

[15] Gmach M.R., Scarpare F.V., Cherubin M.R., Lisboa I.P., Dos Santos A.K.B., Cerri C.E.P., Cerri C.C. (2019). Sugarcane straw removal effects on soil water storage and drainage in southeastern Brazil. J. Soil Water Conserv..

[16] Fortes C., Trivelin P.C.O., Vitti A.C. (2012). Long-term decomposition of sugarcane harvest residues in Sao Paulo state, Brazil. Biomass Bioenergy.

[17] Adalberto C.G., Roberto C.M., Santos M., Martineli S.G., Oliveira B., Carneiro B.L., Junqueira F., Nunes C. (2018). Soil physical quality response to sugarcane straw removal in Brazil: A multi-approach assessment. Soil Tillage Res..

[18] Rossetto R., Cantarella H., Dias F., Landell M., Vitti A.C. (2008). Conservation management and nutrient recycling in sugarcane with a view to mechanical harvesting. Inf. Agronômicas.

[19] Cardoso T.D.F., Cavalett O., Chagas M.F., Morais E.R.D., Carvalho J.L.N., Franco H.C.J., Galdos M.V., Scarpare F.V., Braunbeck O.A., Cortez L.A.B. (2013). Technical and economic assessment of trash recovery in the sugarcane bioenergy production system. Sci. Agric..

[20] Trivelin P.C.O., Franco H.C.J., Otto R., Ferreira D.A., Vitti A.C., Fortes C., Faroni C.E., Oliveira E.C.A., Cantarella H. (2013). Impact of sugarcane trash on fertilizer requirements for São Paulo, Brazil. Sci. Agric..

[21] Magalhães P., Nogueira L., Canatarella H., Rossetto R., Franco H., Braunbeck O.A. (2012). Agro-industrial technological paths. Sustainability of Sugarcane Bioenergy by Center of Strategic Studies and Management.

[22] Berhane M., Xu M., Liang Z., Shi J., Wei G., Tian X. (2020). Effects of long-term straw return on soil organic carbon storage and sequestration rate in North China upland crops: A meta-analysis. Glob. Chang. Biol..

[23] Yang Z.C., Zhao N., Huang F., Lv Y.Z. (2015). Long-term effects of different organic and inorganic fertilizer treatments on soil organic carbon sequestration and crop yields on the North China Plain. Soil Tillage Res..

[24] Liu M., Zhang A., Jiang W., Chen X., Jin R., Zhao P., Tang Z. (2021). Effects of wheat straw incorporation and nitrogen application on sweetpotato yield and quality and soil fertility. Arch. Acker Pflanzenbau Bodenkd..

[25] Han W., He M. (2010). The application of exogenous cellulase to improve soil fertility and plant growth due to acceleration of straw decomposition. Bioresour. Technol..

[26] Shindo H., Nishio T. (2005). Immobilization and remineralization of N following addition of wheat straw into soil: Determination of gross N transformation rates by 15N-ammonium isotope dilution technique. Soil Biol. Biochem..

[27] Muhammad W., Vaughan S.M., Dalal R.C., Menzies N.W. (2011). Crop residues and fertilizer nitrogen influence residue decomposition and nitrous oxide emission from a Vertisol. Biol. Fertil. Soils.

[28] Ludemann C.I., Gruere A., Heffer P., Dobermann A. (2022). Global data on fertilizer use by crop and by country. Sci. Data.

[29] Shah A.N., Iqbal J., Tanveer M., Yang G., Hassan W., Fahad S., Yousaf M., Wu Y. (2017). Nitrogen fertilization and conservation tillage: A review on growth, yield, and greenhouse gas emissions in cotton. Environ. Sci. Pollut. Res..

[30] Tian H., Lu C., Melillo J., Ren W., Huang Y., Xu X., Liu M., Zhang C., Chen G., Pan S. (2012). Food benefit and climate warming potential of nitrogen fertilizer uses in China. Environ. Res. Lett..

[31] Wang Q., Noor H., Sun M., Ren A., Feng Y., Qiao P., Zhang J., Gao Z. (2022). Wide space sowing achieved high productivity and effective nitrogen use of irrigated wheat in South Shanxi, China. PeerJ.

[32] van Wesenbeeck C.F.A., Keyzer M.A., van Veen W.C.M., Qiu H. (2021). Can China’s overuse of fertilizer be reduced without threatening food security and farm incomes?. Agric. Syst..

[33] Mueller N.D., Gerber J.S., Johnston M., Ray D.K., Ramankutty N., Foley J.A. (2012). Closing yield gaps through nutrient and water management. Nature.

[34] Sabir M.S., Shahzadi F., Ali F., Shakeela Q., Niaz Z., Ahmed S. (2021). Comparative Effect of Fertilization Practices on Soil Microbial Diversity and Activity: An Overview. Curr. Microbiol..

[35] Meng H., Xu M., Lv J., He X.H., Wang B., Cai Z. (2014). Quantification of Anthropogenic Acidification Under Long-term Fertilization in the Upland Red Soil of South China. Soil Sci..

[36] Han J., Shi J., Zeng L., Xu J., Wu L. (2017). Impacts of continuous excessive fertilization on soil potential nitrification activity and nitrifying microbial community dynamics in greenhouse system. J. Soil Sediment.

[37] Qiao J., Wang J., Zhao D., Zhu N., Tang J., Zhou W., Schwenke G., Yan T., Yang L. (2022). Effect of continuous N fertilizer reduction on N losses and wheat yield in the Taihu Lake region, China. J. Clean. Prod..

[38] Hu S., Wu Y., Yi N., Zhang S., Zhang Y., Xin X. (2017). Chemical properties of dissolved organic matter derived from sugarcane rind and the impacts on copper adsorption onto red soil. Environ. Sci. Pollut. Res..

[39] Huang J., Duan Y., Xu M., Zhai L., Zhang X., Wang B., Zhang Y., Gao S., Sun N. (2017). Nitrogen mobility, ammonia volatilization, and estimated leaching loss from long-term manure incorporation in red soil. J. Integr. Agric..

[40] Ma Y., Huang G., Shi W., Shi J., Yang B., Lu S., Zhao Q. (2014). Sustainable development of crop farming in Lingchuan County of Guangxi. Acta Ecol. Sin..

[41] Wei D., Zhao X., Wei J., Hu G., Wu X., Cheng M., Wen J., Mo Y. (2022). Effects of Planting Dates on Growth and Yield of Sugarcane in Red Soil. J. Anhui Agric. Sci..

[42] Thuy N.H., Shan Y., Bijay-Singh, Wang K., Cai Z., Yadvinder-Singh, Buresh R.J. (2008). Nitrogen Supply in Rice-Based Cropping Systems as Affected by Crop Residue Management. Soil Sci. Soc. Am. J..

[43] Liu S., Huang D., Chen A., Wei W., Brookes P.C., Li Y., Wu J. (2014). Differential responses of crop yields and soil organic carbon stock to fertilization and rice straw incorporation in three cropping systems in the subtropics. Agric. Ecosyst. Environ..

[44] Fan Y., Gao J., Sun J., Liu J., Su Z., Wang Z., Yu X., Hu S. (2021). Effects of straw returning and potassium fertilizer application on root characteristics and yield of spring maize in China inner Mongolia. Agron. J..

[45] Wei Q., Xu J., Sun L., Wang H., Lv Y., Li Y., Hameed F. (2019). Effects of straw returning on rice growth and yield under water-saving irrigation. Chil. J. Agric. Res..

[46] Yang S., Yaliang L., Weihang X., Lili F., Yihan Z., Linliang L., Xiwen S., Zhian Z. (2012). Effects of Rice Straw Application to Soda-Salinization Paddy Soil on Rice Growth and Grain Yield. Crops.

[47] Fang F.F. (2018). Study on the Effect of Wheat Straw Returning to the Field on the Early Growth of Rice and Its Mechanism. Master’s Thesis.

[48] Geng Y., Cao G., Wang L., Wang S. (2019). Effects of equal chemical fertilizer substitutions with organic manure on yield, dry matter, and nitrogen uptake of spring maize and soil nitrogen distribution. PLoS ONE.

[49] Han J., Dong Y., Zhang M. (2021). Chemical fertilizer reduction with organic fertilizer effectively improve soil fertility and microbial community from newly cultivated land in the Loess Plateau of China. Appl. Soil Ecol..

[50] Bell J.M., Schwartz R., McInnes K.J., Howell T., Morgan C.L.S. (2018). Deficit irrigation effects on yield and yield components of grain sorghum. Agric. Water Manag..

[51] GhassemiSahebi F., Mohammadrezapour O., Delbari M., KhasheiSiuki A., Ritzema H., Cherati A. (2020). Effect of utilization of treated wastewater and seawater with Clinoptilolite-Zeolite on yield and yield components of sorghum. Agric. Water Manag..

[52] Chen Y., Xin L., Liu J., Yuan M., Liu S., Jiang W., Chen J. (2017). Changes in bacterial community of soil induced by long-term straw returning. Sci. Agric..

[53] Mubarak A.R., Rosenani A.B., Anuar A.R., Zauyah S. (2002). Decomposition and nutrient release of maize stover and groundnut haulm under tropical field conditions of malaysia. Commun. Soil Sci. Plant Anal..

[54] Pu J., Jiang N., Zhang Y., Guo L., Huang W., Chen L. (2022). Effects of various straw incorporation strategies on soil phosphorus fractions and transformations. Gcb Bioenergy.

[55] Wei K., Chen Z., Jiang N., Zhang Y., Feng J., Tian J., Chen X., Lou C., Chen L. (2021). Effects of mineral phosphorus fertilizer reduction and maize straw incorporation on soil phosphorus availability, acid phosphatase activity, and maize grain yield in northeast China. Arch. Acker Pflanzenbau Bodenkd..

[56] Damon P.M., Bowden B., Rose T., Rengel Z. (2014). Crop residue contributions to phosphorus pools in agricultural soils: A review. Soil Biol. Biochem..

[57] Li Y., Wang J., Shao M. (2022). Earthworm inoculation and straw return decrease the phosphorus adsorption capacity of soils in the Loess region, China. J. Environ. Manag..

[58] Meier E.A., Thorburn P.J., Wegener M.K., Basford K.E. (2006). The availability of nitrogen from sugarcane trash on contrasting soils in the wet tropics of North Queensland. Nutr. Cycl. Agroecosystems.

[59] Yan Y., Ji W., Li B., Wang G., Chen S., Zhu D., Liu Z. (2022). Quantification of the effects of long-term straw return on soil organic matter spatiotemporal variation: A case study in typical black soil region. EGUsphere.

[60] Yan Y., Ji W., Li B., Wang G., Hu B., Zhang C., Mouazen A.M. (2022). Effects of Long-Term Straw Return and Environmental Factors on the Spatiotemporal Variability of Soil Organic Matter in the Black Soil Region: A Case Study. Agronomy.

[61] Li Z., Li D., Ma L., Yu Y., Zhao B., Zhang J. (2019). Effects of straw management and nitrogen application rate on soil organic matter fractions and microbial properties in North China Plain. J. Soils Sediments.

[62] Guo Z., Liu H., Hua K., Wang D., He C. (2018). Long-term straw incorporation benefits the elevation of soil phosphorus availability and use efficiency in the agroecosystem. Span. J. Agric. Res..

[63] Li X.G., Jia B., Lv J., Ma Q., Kuzyakov Y., Li F. (2017). Nitrogen fertilization decreases the decomposition of soil organic matter and plant residues in planted soils. Soil Biol. Biochem..

[64] Pittelkow C.M., Liang X., Linquist B.A., van Groenigen K.J., Lee J., Lundy M.E., van Gestel N., Six J., Venterea R.T., van Kessel C. (2015). Productivity limits and potentials of the principles of conservation agriculture. Nature.

[65] Poeplau C., Kätterer T., Bolinder M.A., Börjesson G., Berti A., Lugato E. (2015). Low stabilization of aboveground crop residue carbon in sandy soils of Swedish long-term experiments. Geoderma.

[66] Powlson D.S., Glendining M.J., Coleman K., Whitmore A.P. (2011). Implications for Soil Properties of Removing Cereal Straw: Results from Long-Term Studies1. Agron. J..

[67] Zhang G., Zhang W., Pu X., Zhang P., Ou Y. (2019). Influence of Stalk Residue Retention and Fertilization on the Rhizospheric Effect with Drip-Irrigated Cotton. Agron. J..

[68] Ran C., Gao D., Liu W., Guo L., Bai T., Shao X., Geng Y. (2022). Straw and nitrogen amendments improve soil, rice yield, and roots in a saline sodic soil. Rhizosphere.

[69] Song X., Sun R., Chen W., Wang M. (2020). Effects of surface straw mulching and buried straw layer on soil water content and salinity dynamics in saline soils. Can. J. Soil. Sci..

[70] Tan Y., Li H., Xu S., Liu B., Jiang Z., Wei G. (2010). Effects of Cane Leave Residue on the Soil Fertility. Sugar Crops China.

[71] Liao Q., Wei G., Chen G., Liu B., Huang D., Li Y. (2014). Effect of Trash Addition to the Soil on Microbial Communities and Physico-Chemical Properties of Soils and Growth of Sugarcane Plants. Sugar Tech.

[72] Wuest S.B. (2009). Correction of Bulk Density and Sampling Method Biases Using Soil Mass per Unit Area. Soil Sci. Soc. Am. J..

[73] Mckee G.W. (1964). A Coefficient for Computing Leaf Area in Hybrid Corn1. Agron. J..

[74] Bu L., Liu J., Zhu L., Luo S., Chen X., Li S., Lee Hill R., Zhao Y. (2013). The effects of mulching on maize growth, yield and water use in a semi-arid region. Agric. Water Manag..

